# Subcortical brain alterations in major depressive disorder: findings from the ENIGMA Major Depressive Disorder working group

**DOI:** 10.1038/mp.2015.69

**Published:** 2015-06-30

**Authors:** L Schmaal, D J Veltman, T G M van Erp, P G Sämann, T Frodl, N Jahanshad, E Loehrer, H Tiemeier, A Hofman, W J Niessen, M W Vernooij, M A Ikram, K Wittfeld, H J Grabe, A Block, K Hegenscheid, H Völzke, D Hoehn, M Czisch, J Lagopoulos, S N Hatton, I B Hickie, R Goya-Maldonado, B Krämer, O Gruber, B Couvy-Duchesne, M E Rentería, L T Strike, N T Mills, G I de Zubicaray, K L McMahon, S E Medland, N G Martin, N A Gillespie, M J Wright, G B Hall, G M MacQueen, E M Frey, A Carballedo, L S van Velzen, M J van Tol, N J van der Wee, I M Veer, H Walter, K Schnell, E Schramm, C Normann, D Schoepf, C Konrad, B Zurowski, T Nickson, A M McIntosh, M Papmeyer, H C Whalley, J E Sussmann, B R Godlewska, P J Cowen, F H Fischer, M Rose, B W J H Penninx, P M Thompson, D P Hibar

**Affiliations:** 1 Department of Psychiatry and Neuroscience Campus Amsterdam, VU University Medical Center, Amsterdam, The Netherlands; 2 Department of Psychiatry and Human Behavior, University of California, Irvine, CA, USA; 3 Max Planck Institute of Psychiatry, Munich, Germany; 4 Department of Psychiatry, University of Regensburg, Regensburg, Germany; 5 Department of Psychiatry, University of Dublin, Trinity College, Dublin, Ireland; 6 Imaging Genetics Center, Department of Neurology, Keck School of Medicine, University of Southern California, Marina del Rey, CA, USA; 7 Department of Epidemiology, Erasmus MC University Medical Center, Rotterdam, The Netherlands; 8 Department of Psychiatry, Erasmus MC University Medical Center, Rotterdam, The Netherlands; 9 Departments of Radiology and Medical Informatics, Erasmus MC University Medical Center, Rotterdam, The Netherlands; 10 Imaging Science and Technology, Faculty of Applied Sciences, Delft University of Technology, Delft, The Netherlands; 11 Department of Neurology, Erasmus MC University Medical Center, Rotterdam, The Netherlands; 12 German Center for Neurodegenerative Diseases (DZNE), Rostock/Greifswald, Germany; 13 Department of Psychiatry and Psychotherapy, University Medicine Greifswald, Greifswald, Germany; 14 Helios Hospital Stralsund, Stralsund, Germany; 15 Institute of Diagnostic Radiology and Neuroradiology, University Medicine Greifswald, Greifswald, Germany; 16 Institute for Community Medicine, University Medicine Greifswald, Greifswald, Germany; 17 Clinical Research Unit, Brain and Mind Research Institute, University of Sydney, Camperdown, Australia; 18 Center for Translational Research in Systems Neuroscience and Psychiatry, Department of Psychiatry and Psychotherapy, University Medical Center, Goettingen, Germany; 19 NeuroImaging Genetics, QIMR Berghofer Medical Research Institute, Brisbane, QLD, Australia; 20 School of Psychology, University of Queensland, Brisbane, QLD, Australia; 21 Center for Advanced Imaging, University of Queensland, Brisbane, QLD, Australia; 22 Genetic Epidemiology, QIMR Berghofer Medical Research Institute, Brisbane, QLD, Australia; 23 Queensland Brain Institute, University of Queensland, Brisbane, QLD, Australia; 24 Quantitative Genetics, QIMR Berghofer Medical Research Institute, Brisbane, QLD, Australia; 25 Virginia Institute for Psychiatric and Behavioral Genetics, Virginia Commonwealth University, Richmond, VA, USA; 26 Department of Psychology, Neuroscience and Behaviour, McMaster University, Hamilton, ON, Canada; 27 Department of Psychiatry, Mathison Centre for Mental Health Research and Education, Hotchkiss Brain Institute, Cumming School of Medicine, University of Calgary, Calgary, AB, Canada; 28 Department of Psychiatry and Institute of Neuroscience, University of Dublin, Trinity College Dublin, Dublin, Ireland; 29 University of Groningen, University Medical Center Groningen, NeuroImaging Center, Groningen, The Netherlands; 30 Department of Psychiatry, Leiden University Medical Center, Leiden University, Leiden, The Netherlands; 31 Leiden Institute for Brain and Cognition, Leiden, The Netherlands; 32 Department of Psychiatry and Psychotherapy, Division of Mind and Brain Research, Charité Universitätsmedizin Berlin, Berlin, Germany; 33 Department of General Psychiatry, University Hospital Heidelberg, Heidelberg, Germany; 34 Department of Psychiatry and Psychotherapy, University Medical Center Freiburg, Freiburg im Breisgau, Germany; 35 Department of Psychiatry, University of Bonn, Bonn, Germany; 36 Department of Psychiatry and Psychotherapy, Philipps-University Marburg, Marburg, Germany; 37 Center for Integrative Psychiatry, University of Lübeck, Lübeck, Germany; 38 Division of Psychiatry, University of Edinburgh, Edinburgh, UK; 39 Centre for Cognitive Ageing and Cognitive Epidemiology, University of Edinburgh, Edinburgh, UK; 40 University Department of Psychiatry, Warneford Hospital, Oxford, UK; 41 Department of Psychosomatic Medicine, Center for Internal Medicine and Dermatology, Charité Universitätsmedizin, Berlin, Germany; 42 Institute for Social Medicine, Epidemology and Health Economics, Charité Universitätsmedizin, Berlin, Germany; 43 Department of Quantitative Health Sciences, University of Massachusetts Medical School, Worcester, MA, USA

## Abstract

The pattern of structural brain alterations associated with major depressive disorder (MDD) remains unresolved. This is in part due to small sample sizes of neuroimaging studies resulting in limited statistical power, disease heterogeneity and the complex interactions between clinical characteristics and brain morphology. To address this, we meta-analyzed three-dimensional brain magnetic resonance imaging data from 1728 MDD patients and 7199 controls from 15 research samples worldwide, to identify subcortical brain volumes that robustly discriminate MDD patients from healthy controls. Relative to controls, patients had significantly lower hippocampal volumes (Cohen's *d*=−0.14, % difference=−1.24). This effect was driven by patients with recurrent MDD (Cohen's *d*=−0.17, % difference=−1.44), and we detected no differences between first episode patients and controls. Age of onset ⩽21 was associated with a smaller hippocampus (Cohen's *d*=−0.20, % difference=−1.85) and a trend toward smaller amygdala (Cohen's *d*=−0.11, % difference=−1.23) and larger lateral ventricles (Cohen's *d*=0.12, % difference=5.11). Symptom severity at study inclusion was not associated with any regional brain volumes. Sample characteristics such as mean age, proportion of antidepressant users and proportion of remitted patients, and methodological characteristics did not significantly moderate alterations in brain volumes in MDD. Samples with a higher proportion of antipsychotic medication users showed larger caudate volumes in MDD patients compared with controls. This currently largest worldwide effort to identify subcortical brain alterations showed robust smaller hippocampal volumes in MDD patients, moderated by age of onset and first episode versus recurrent episode status.

## Introduction

With a lifetime prevalence of >16%,^1^ major depressive disorder (MDD) is a very common psychiatric disease and is among the leading causes of disability worldwide.^2^ Despite intensive research aimed at identifying neurobiological substrates of depression in the last decades, our understanding of the pathophysiological mechanisms underlying depression is still rudimentary. Widely available structural magnetic resonance imaging has led to hypotheses of (para-)limbic circuits being involved in MDD, but still the exact pattern of structural brain alterations associated with MDD remains unresolved, perhaps due to small sample sizes, disease heterogeneity and the complex interactions between clinical characteristics and brain morphology. Therefore, in the current study we meta-analyzed structural magnetic resonance imaging data of a very large sample (*n*=8927) to identify subcortical brain volumes that robustly discriminate MDD patients from healthy controls.

Many studies have found structural alterations in various subcortical brain regions in MDD. To date, volumetric differences have not always been consistent and are poorly replicated for some regions; moreover, sample sizes are often small, limiting the power to detect subtle brain differences. Meta–analyses represent useful tools to identify the most robust findings across studies, and indicate that morphological changes in MDD are regional rather than global.^3, 4, 5^ Hippocampal volume reduction (one of the most extensively studied regions) in MDD has been one of the most widely replicated findings.^3^ However, the association between MDD and hippocampal volume reduction is likely complex, and hippocampal volumes may be smaller, on average, in patients with recurrent MDD compared to patients early in the course of adult-onset MDD.^6^ Other factors may modulate the effect size of morphological changes in the hippocampus in MDD, including disease severity, childhood maltreatment, age of onset, antidepressant medication and illness duration.^6, 7^

Amygdala volume abnormalities in MDD are inconsistently reported and may depend on the illness phase, medication use and family history of MDD. A current first episode and antidepressant use has been associated with enlarged amygdala volume (for example, van Eijndhoven *et al.*,^8^ Frodl *et al.*^9^ and Hamilton *et al.*^10^), but a greater number of episodes and a family history of MDD have been associated with smaller amygdala volume (for example, Hronenberg *et al.*^11^ and Saleh *et al.*^12^). Other brain regions have been less extensively investigated, including prefrontal areas, thalamus and striatum.

Sources of inconsistency in prior findings are multifactorial. Demographic characteristics of the samples such as age and sex may partly explain differences in effect sizes across studies. Clinical characteristics of MDD samples are another major source of heterogeneity. Moreover, differences in data acquisition protocols and processing, differences in statistical analyses performed and potential publication bias further complicate the interpretation of findings from retrospective meta-analyses. For instance, different automated or manual segmentation algorithms may show subtle differences in their estimates of subcortical volumes.^13^

To address the limited statistical power of prior studies and overcome some of the issues of retrospective meta-analyses based on aggregated results from single studies, we initiated the Major Depressive Disorder Working Group within the Enhancing Neuro Imaging Genetics through Meta-Analysis (ENIGMA) consortium. This is an international collaboration currently evaluating 15 research samples from six different countries worldwide, including neuroimaging data from 1728 MDD patients and 7199 healthy individuals. The primary aim of our ENIGMA-MDD Working Group is to identify imaging markers that robustly discriminate MDD patients from healthy controls using an individual participant data (IPD)-based meta-analysis. This IPD-based meta-analytic approach applied to data from our consortium provided us with the opportunity to plan *a priori*, standardize and harmonize data processing and statistical models across all samples, and to assess the influence of participant-level covariates, not all of which are reported in the literature, thereby addressing some of the limitations related to retrospective meta-analyses. Imaging markers identified through our consortium could help to prioritize brain measures for future genetic analyses, and may enhance our understanding of the etiology of MDD.

Here, we investigated subcortical gray matter, lateral ventricle and total intracranial volume (ICV) alterations in MDD patients compared to healthy individuals by performing the largest meta-analysis to date. As additional exploratory analyses, we examined potential modulating effects of demographic, clinical and methodological characteristics on morphological differences in MDD patients.

## Subjects and methods

### Samples

The ENIGMA-MDD Working Group includes 15 international samples with neuroimaging and clinical data from MDD patients and healthy controls (participating sites are shown in Supplementary Figure S1). None of the research groups that we approached refused to participate in the ENIGMA-MDD consortium. Moreover, we are continuously encouraging new research groups to join in on our ongoing consortium work to increase our sample size and thereby increase statistical power and generalizability of our results. Detailed demographics for each sample are found in Supplementary Table S1 and clinical characteristics in Supplementary Table S2. Exclusion criteria for study enrollment in each sample are given in Supplementary Table S3. In total, we analyzed data from 8927 people including 1728 MDD patients and 7199 healthy controls. All participating sites obtained approval from local institutional review boards and ethics committees. All study participants provided written informed consent at their local institution.

### Image processing and analysis

Structural T1-weighted magnetic resonance imaging brain scans were acquired at each site and analyzed locally using the fully automated and validated segmentation software FreeSurfer.^14^ Image acquisition parameters and software descriptions for each sample are given in Supplementary Table S4. The segmentations of seven subcortical gray matter regions (nucleus accumbens, amygdala, caudate, hippocampus, pallidum, putamen and thalamus), lateral ventricles and total ICV were visually inspected for accuracy following standardized protocols designed to facilitate harmonized image analysis across multiple sites (http://enigma.ini.usc.edu/protocols/imaging-protocols/). Further details on image exclusion criteria and quality control can be found in Supplementary Information SI1.

### Statistical framework of meta-analysis

We examined patient versus control group differences within each sample using multiple linear regression models, where the mean ((left+right)/ 2) region of interest volume was the outcome measure and a binary indicator of diagnosis (0=controls, 1=patients) was the predictor of interest. All models were controlled for age, sex and ICV. Additional covariates were included whenever necessary to control for scanner differences. Effect size estimates were calculated using Cohen's *d*-metric computed from the *t*-statistic of the diagnosis indicator variable from the regression models. Throughout the manuscript we report uncorrected *P*-values with a significance threshold determined by Bonferroni correction for testing nine regions of interest (*P*=0.05/9=5.6 × 10^−3^).

To explore the influence of sex on subcortical brain volume differences between patients and controls, we performed diagnosis-by-sex interaction effects within each sample. To further investigate the sources of regional brain changes in MDD, we performed separate stratified meta-analyses comparing age of onset (early onset ⩽21 years, late onset >21 years^15^) and stage of illness (first and recurrent episode patients). Further, we investigated whether symptom severity at the time of scanning was associated with brain changes using the 17-item Hamilton Depression Rating Scale (HDRS-17^16^) and the Beck Depression Inventory (BDI-II^17^).

All regression models and effect size estimates were fit at each site separately and a final Cohen's *d*-effect size estimate was obtained using an inverse variance-weighted random-effect meta-analysis model in R (metafor package, version 1.9-1^18^). The meta-analysis of symptom severity scores was an exception: severity scores were treated as continuous variables, so effect sizes were in terms of Pearson's *r,* a partial-correlation after removing nuisance variables (age, sex, ICV and scan center). The final meta-analyzed Pearson's *r* was estimated following the same inverse variance-weighted random-effect meta-analysis model used for all other meta-analyses. See Supplementary Information SI1 for full meta-analysis details.

### Moderator analyses with meta-regression

We tested whether mean age of each sample (Supplementary Table S1), magnetic resonance field strength, FreeSurfer version used for image processing (Supplementary Table S4), percentage of patients acutely depressed, percentage of patients with a co-occurring anxiety disorder, percentage of patients taking antidepressants and the percentage of patients taking antipsychotics (Supplementary Table S2) explained a significant proportion of the variance in effect sizes across sites in the meta-analysis. Each moderator variable was separately included as a fixed effect predictor in a meta-regression model.

## Results

### Effect sizes for group differences in subcortical volumes

#### Comparison of brain volumes in MDD patients and healthy controls

**Figure 1:**
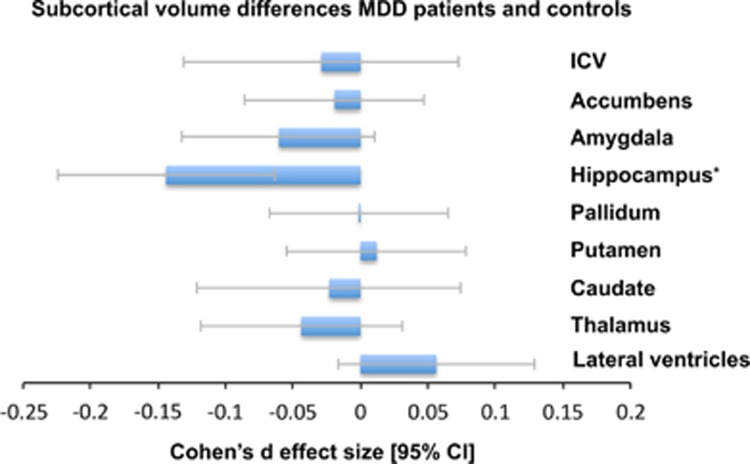
Cohen's *d-*effect sizes 95% CI and for differences in subcortical brain volumes between major depressive disorder (MDD) patients and healthy control subjects. Effect sizes were corrected for age, sex and intracranial volume (ICV). The effect size for ICV was corrected for age and sex. *P<0.05 corrected. CI, confidence interval.

In our primary analysis, we assessed case-control differences between all MDD patients (*n*=1728) compared with all healthy controls (*n*=7199) across nine brain structures (Figure 1). Only mean hippocampal volume was significantly lower in MDD patients compared to healthy controls (Cohen's *d* (95% confidence interval): *d*=−0.14 (−0.22, −0.06); *P*-value=4.60 × 10^−4^, % difference=−1.25). Case-control differences for all structures are listed in Table 1. No significant diagnosis-by-sex interaction effects on any of the region of interest volumes were observed (Supplementary Table S16).

#### Influence of recurrence status on brain volume

**Figure 2:**
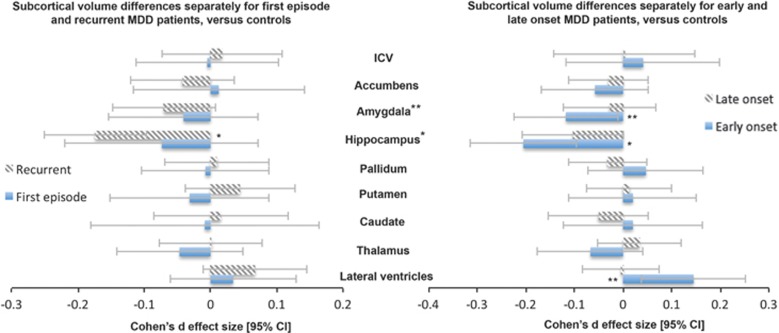
(**a**) Cohen's *d*-effect sizes 95% CI for differences in subcortical brain volumes between recurrent major depressive disorder (MDD) patients and healthy control subjects (striped pattern) and between first episode MDD patients and healthy controls (no pattern). (**b**) Cohen's *d*-effect sizes 95% CI for differences in subcortical brain volumes between early onset (⩽21) MDD patients and healthy control subjects (no pattern) and between later onset (>21) MDD patients and healthy controls (striped pattern). Effect sizes were corrected for age, sex and intracranial volume (ICV). *P<0.05 corrected, **P<0.05. CI, confidence interval.

We examined how the current stage of a depressed patient may relate to brain volumes by splitting the sample into first episode (*n*=583) and recurrent episode patients (*n*=1119) and compared to healthy controls (Figure 2a). We did not detect any significant differences between first episode patients and healthy controls (all *P*-values >0.3). Recurrent episode patients showed lower mean hippocampal volume than controls (*d*=−0.17 (−0.25, −0.10); *P*-value=1.12 × 10^−5^, % difference=−1.44). Full meta-analyzed recurrence status differences are listed in Supplementary Table S5 and Supplementary Table S6. Relative to the full MDD sample, recurrent patients showed larger effect sizes compared to controls. However, no significant differences were detected between recurrent and first episode patients (Supplementary Table S7).

#### Influence of age of onset of depression on brain volume

We examined how the age of onset modulates volumetric brain changes (Figure 2b). Patients with an early age of onset (⩽21 years; *n*=541) showed significantly lower mean hippocampal volumes than controls (*d*=−0.20 (−0.31, −0.10); *P*-value=2.31 × 10^−4^, % difference=−1.85). In addition, we found lower amygdala volume (*d*=−0.12 (−0.23, −0.01); *P*-value=0.033, % difference=−1.23) and higher mean lateral ventricle volume (*d*=0.14 (0.04, 0.25); *P*-value=0.009, % difference=5.11) in early onset patients compared to controls, but neither survived correction for multiple comparisons. Although sample sizes were too small to split first and recurrent episode patients into early and late onset groups, only about half (57%) of the early onset MDD patients had a recurrent episode, and the percentage of recurrent episode patients did not moderate the result of smaller hippocampal volumes in early onset MDD patients (*P*=0.54), suggesting that this effect is at least partly independent of recurrence status. Patients with a late age of onset (>21 years; *n*=997) showed no detectable brain volumetric differences compared to controls. Full age of onset effect sizes are listed in Supplementary Table S8 and Supplementary Table S9. The effect sizes in early episode patients are larger than those obtained when considering the full sample. However, there were no detectable differences between early and late onset patients (see Supplementary Table S10).

### Association of MDD symptoms severity with subcortical volumes

We did not detect any significant associations between symptom severity at study inclusion and brain volumes using the HDRS-17 (*n*=667) and BDI-II (*n*=667) questionnaires. Full effect sizes of severity scores are available in Supplementary Table S11 and Supplementary Table S12.

### Moderator analyses

Using meta-regression, we tested whether hypothesized moderating factors influenced the effect size estimates of all brain volumes across samples included in the meta-analysis. Mean age of each sample, magnetic resonance field strength, percent of patients with a co-occurring anxiety disorder, and percent of acutely depressed patients in each sample did not moderate differences for subcortical volumes. We found an influence of the FreeSurfer version used at each site on amygdala volume (β=−0.31; *P*-value=0.049), but this was not significant after correcting for multiple comparisons. We also found an influence of percentage of patients taking antidepressants on hippocampal effect size estimates (β=−0.0023; *P*-value=0.038), but again this was not significant after correction for multiple comparisons. Finally, we found a near-significant effect of antipsychotic drug use on mean caudate volume (β=0.016; *P*-value=6.71 × 10^−3^), implying that caudate volume of depressed patients was greater as the percentage of patients taking antipsychotic drugs was higher (Supplementary Figure S7). Full results from each of the moderator analyses are available in Supplementary Table S15.

### Power analysis

With 1728 MDD patients and 7199 controls, we were able to detect brain volume differences as small as Cohen's *d*=0.0751 at a nominal significance level *P*-value=0.05 and 80% power (and Cohen's *d*=0.0968 at our study significance threshold *P*-value=5.6 × 10^−3^). On the basis of the final meta-analyzed effect sizes found in this study, to detect mean differences in hippocampal volumes between MDD patients and controls, a study in the general MDD population at large would require 802 subjects per group to have 80% power to detect a difference at *P*-value=0.05. When focusing analyses on recurrent MDD patients and controls, 545 subjects per group would be needed to provide 80% power to detect a difference in hippocampal volume at *P*-value=0.05. When focusing analyses on early age of onset MDD patients (⩽21 years) and controls, 394 subjects per group would be needed to have 80% power to detect a difference in hippocampal volume at *P*-value=0.05. See Supplementary Information SI2 for full details of the power analysis.

## Discussion

This worldwide effort to identify subcortical gray matter, lateral ventricle and total ICV alterations associated with MDD using an IPD-based meta-analytic approach showed robust reductions in hippocampal volume (1.24%) in MDD patients compared with healthy controls. These hippocampal volume reductions were mainly present in recurrent and/or early onset (⩽21 years) MDD, whereas hippocampal volume reductions were absent in first episode patients and less pronounced in patients with later age of onset (>21 years) MDD. Furthermore, smaller amygdala volume and larger lateral ventricles volume were found in early onset MDD, but neither survived correction for multiple comparisons.

Our finding of smaller hippocampal volume in MDD is in line with previous retrospective meta-analyses of aggregated data.^3, 4, 5, 6^ This robust finding of smaller hippocampal volume is often linked to the ‘neurotrophic hypothesis of depression'. This proposes that elevated glucocorticoid levels associated with chronic hyperactivity of the hypothalamic–pituitary–adrenal axis in MDD may induce brain atrophy via remodeling and downregulation of growth factors including brain-derived neurotrophic factor.^19^ This process may preferentially target the hippocampus, a major site in the glucocorticoid negative feedback loop of the hypothalamic–pituitary–adrenal axis with high expression of glucocorticoid receptors.^20^ In animal models of depression, stress-induced increases in glucocorticoid levels may result in regression of dendritic processes, inhibition of neurogenesis and loss of neurons.^21^ We only observed hippocampal volume deficits in recurrent MDD and not in first episode patients. There is also direct evidence from longitudinal studies that hippocampal volume progressively decreases during the course of the disease, beyond levels expected from normal aging.^22^ We also found that hippocampal volume reductions were more pronounced in early onset patients (⩽21 years), consistent with prior findings of smaller hippocampal volume in early onset depression.^23^ Several factors, including early life stress, temperamental low effortful control and family history of depression have been linked to early onset depression,^24, 25, 26^ as well as smaller hippocampal volume.^27, 28^

Patterns of abnormal hippocampal development may predate adolescent depression onset^29^ and smaller hippocampal volumes have been found to predict a protracted response to antidepressant treatment over weeks^30, 31^and a more severe long-term illness course,^32^ so morphological hippocampal alterations may represent risk markers for depression, recurrence and chronicity. As we only observed hippocampal volume reduction in recurrent patients and no subcortical volume alterations in first episode patients, the current finding of smaller hippocampal volume in early onset depression may reflect a longer illness duration and/or greater number of episodes in patients with early onset MDD instead of a premorbid vulnerability factor. Nonetheless, as only about half (57%) of the early onset patients in our meta-analysis had a recurrent episode and as the percentage of recurrent episode patients did not moderate the result of smaller hippocampal volumes in early onset patients (*post hoc* test, *P*=0.54), we conclude that early disease onset is (in part) independently associated with lower hippocampal volumes. Unfortunately, for many of the samples complete information on the exact number of episodes and duration of each episode was not available and simply calculating disease burden as current age minus age of onset is unsatisfactory, given the relapsing and remitting nature of MDD. Clearly, there is a continued need for longitudinal studies tracking hippocampal volume changes over the disease course, to further elucidate whether hippocampal abnormalities result from prolonged duration of chronic stress (that is, ‘scarring‘), represent a vulnerability factor for MDD, or both.

Prior structural neuroimaging studies in depression have yielded conflicting results regarding alterations in amygdala volume (for example, van Eijndhoven *et al.*,^8^ Frodl *et al.*,^9^ Kronenberg *et al.*^11^ and Hickie *et al.*^33^). Our meta-analysis suggested smaller amygdala volume in early onset MDD, but this effect was not significant after correction for multiple comparisons. This is consistent with a previous retrospective meta-analysis of aggregated amygdala volume data by Hamilton *et al.,*^10^ which also failed to detect a reliable difference in amygdala volume between depressed patients and controls. However, prior meta-analyses have shown larger amygdala volume in samples with a higher proportion of patients taking antidepressants.^3, 10^ We could not replicate this result in the current meta-analysis.

Furthermore, our controlled meta-analysis could not replicate findings of smaller caudate and putamen volumes observed in prior smaller meta-analyses.^3, 5^ Our current observations suggest that subcortical alterations in MDD are either very small or limited to the hippocampus (and to a lesser extent the amygdala) rather than widespread. Reduced hippocampal volume is not specific to MDD, as it has been observed in other psychiatric disorders, such as schizophrenia, posttraumatic stress disorder, borderline personality disorder and obsessive-compulsive disorder.^34^ Interestingly, IPD-based meta-analytic studies of both schizophrenia^35^ and bipolar disorder (Hibar *et al.*, submitted), using highly similar protocols, reported more widespread alterations in subcortical volumes. Generally, detrimental effects of schizophrenia and bipolar disorder on subcortical brain volumes appear greater than those in MDD, although a study design directly comparing patients with these disorders is clearly needed to resolve this.

The current study is the first to meta-analyze the relationship between symptom severity at study inclusion and subcortical volumes in MDD patients. Previous studies have yielded conflicting results; most studies found no correlations between depressive symptom severity and brain volume (for example, Kronenberg *et al.*,^11^ Hickie *et al.*^36^ and Frodl *et al.*^37^), but some studies found positive^8, 38^ as well as inverse associations^39^ with, for example, amygdala and hippocampal volumes. Our meta-analyses, based on the HDRS-17 (*n*=667) and BDI-II (*n*=667) questionnaires, found no evidence of association between symptom severity and subcortical gray matter volumes. As severity of an entire depressive episode is not fully characterized by depression severity at study inclusion and given the predictive value of hippocampal volume,^30, 31, 32^ this result awaits further investigation in prospective longitudinal treatment designs.

With respect to the moderating effects of medication use, no significant effect of the percentage of patients taking antidepressants was observed, only a trend-wise lower hippocampal volume in MDD patients in samples with a higher percentage of patients taking antidepressants. Antidepressant treatment may block hippocampal atrophy, to some extent, by enhancing synaptic plasticity, neurotrophic processes and putatively neurogenesis,^40^ which appears to contradict our current observation. However, confounding interactions between antidepressant use and clinical characteristics cannot be ruled out, as samples with a higher percentage of antidepressant users are likely to include more severe MDD patients. Indeed, when performing a *post hoc* analysis, the trend effect of lower hippocampal volume in antidepressant medication users compared to controls disappeared when controlling for recurrence in the regression model. However, intervention studies with pre- and post-antidepressant treatment comparisons are needed to clarify the impact of antidepressant use on hippocampal volume.

We found that an increase in the percentage of patients taking antipsychotic drugs was associated with larger caudate volume in depressed patients relative to controls. This is in line with prior observations of caudate enlargement following antipsychotic treatment in patients with schizophrenia and can occur as early as 3 weeks post-exposure^41, 42^ (but see Crespo-Facorro *et al.*^43^).

A key strength of our study is the IPD meta-analytic approach, which increased the power to detect small effects by combining data from 8927 individuals, while ensuring low methodological heterogeneity by standardizing brain segmentation techniques and statistical models across all participating samples. Moreover, this IPD-based approach allowed us to systematically investigate the effects of clinical characteristics such as recurrence status, age of onset and severity of symptoms on brain alterations in MDD patients.

Nonetheless, our study has some limitations. First, we could not directly examine the influence of remission of MDD on brain volume. In most studies, the majority of patients were acutely depressed at the time of scanning, yielding groups of remitted patients that were too small to properly investigate how remission relates to brain volume. However, the proportion of MDD patients in remission at each site had no moderating effect on differences in brain volume when examined in a meta-regression analysis (see Supplementary Table S15). Moreover, an additional meta-analysis including only samples with a current diagnosis of MDD patients at the time of scanning revealed very similar results (see Supplementary Table S13), so remission status presumably did not have a major impact on our current findings. A second limitation is the variety of questionnaires used by the different sites to assess severity of depressive symptoms; scores from different studies were not directly comparable. Because pooling results from different severity measures in a meta-analysis may lead to biased results,^44^ we limited the meta-analysis with respect to severity of symptoms to a selection of studies using the same instrument, which considerably reduced the sample size. Finally, we restricted our analyses to subcortical gray matter, lateral ventricle and total ICVs and did not include cortical measures. As efforts such as the current one require significant work in harmonization and quality control by all sites, the current study is only a first step toward identifying robust brain volume alterations in MDD patients. Because prior studies have shown a potentially important role of especially the anterior cingulate cortex,^45^ the important next step within our consortium will be examining cortical brain alterations associated with MDD.

Despite these limitations, results of this first initiative of the ENIGMA-MDD working group clearly indicate a key role of the hippocampus in the pathophysiology of MDD, showing robust hippocampal volume reductions particularly in recurrent and early onset MDD. Brain changes in other subcortical regions in MDD were less evident. Our findings together with the observations of associations between smaller hippocampal volume and executive impairments,^37^ learning and memory deficits^36^ and worse treatment response^46^ in MDD, suggest that the hippocampus is a prime target region for future research aimed at further unraveling the pathophysiology of MDD and improving treatment.

## Figures and Tables

**Table 1:** Full meta-analytic results for each mean structure for the MDD patients versus controls comparison controlling for age, sex, scan center and ICV

	*Cohen's d^a^* *(MDD—CTL)*	*Standard error*	*95% CI*	*% Difference*	P*-value*	*I*^*2*^	*Number of controls*	*Number of patients*
Lateral ventricles	0.056	0.037	−0.017–0.129	1.345	0.130	12.162	7058	1689
Thalamus	−0.044	0.038	−0.119–0.031	−0.398	0.250	15.067	7046	1682
Caudate	−0.023	0.050	−0.121–0.074	−0.232	0.641	45.729	7034	1681
Putamen	0.012	0.034	−0.054–0.078	0.104	0.722	0.005	6957	1656
Pallidum	−0.001	0.034	−0.067–0.065	−0.049	0.972	0.000	7018	1657
Hippocampus	−0.144	0.041	−0.225 to −0.064	−1.245	4.60 × 10^−4^	24.813	7040	1700
Amygdala	−0.060	0.036	−0.132–0.011	−0.658	0.097	10.152	7060	1696
Accumbens	−0.019	0.034	−0.085–0.047	−0.203	0.569	0.000	6967	1652
ICV	−0.029	0.052	−0.131–0.073	−0.212	0.575	51.476	7199	1728

Abbreviations: CI, confidence intervals; CTL, control; ICV, intracranial volume; MDD, major depressive disorder.

a Included samples: all samples.

Adjusted Cohen's *d* is reported.
