# Supplementary material for: Subcortical brain alterations in major depressive disorder: findings from the ENIGMA Major Depressive Disorder working group
Source: Mol Psychiatry. 2015 Jun 30;21(6):806–12. doi: 10.1038/mp.2015.69 (PMC4879183; doi:10.1038/mp.2015.69)
Supplement: Supplementary Information [file mp201569x1.doc]

Supplementary Information

**Supplementary Information SI1:**

Image Exclusion Criteria:

Each image segmentation was individually examined by a neuroimaging expert at each site by overlaying the segmentation label of each structure on the T1-weighted brain scan. Further, we collected study-wide statistics (means and standard deviations) as well as histogram plots in order to identify non-normally distributed data and major outliers. A subject was considered a statistical outlier if its volume was >2.698 standard deviations away from the global mean. For each subject that was marked as a statistical outlier, individual sites were asked to re-inspect the subject’s segmentation in order to verify that it was properly segmented. If a subject was a statistical outlier, but was properly segmented it was kept in the analysis. Otherwise the subject was removed.

Age at Illness Onset:

Four samples were not included in the early age of onset analysis because of missing information on age of onset (CODE, Rotterdam study) or too small sample sizes (Clinical Depression Dublin N=7, Edinburgh N=6), see **Table S8**. Four samples were not included in the late age of onset analysis because of missing information (CODE, Rotterdam study) or too small sample sizes (Edinburgh N=9, QTIM N=6), see **Table S9**. Patients with early and late age of onset were separately compared to controls and then with each other. Illness stage analyses split patients into first-episode patients and recurrent-episode patients, which were separately compared with controls and then with each other. Three samples were excluded from the first episode patients analysis because of missing information on recurrence (Edinburgh) or too small sample sizes (CODE N=0, Imaging Genetics Dublin N=8), see **Table S5**. One sample was not included in the recurrent episode analysis because of missing information (Edinburgh), see **Table S6**.

Severity Analyses:

Unfortunately, not all sites used the same symptom severity measurements, with nine sites reporting HDRS-17 measurements and four sites reporting BDI-II (see **Table S11 and S12**). One additional site (SHIP-trend) used the Patient Health Questionnaire (PHQ-9), but the total score was converted to a BDI-II total score on basis of a common metric developed for 11 depression questionnaires including the PHQ-9 and BDI-II by Wahl et al. (1). Symptom severity scores were analyzed separately as combining them could provide biased estimates of effects (2) and only three sites had severity scores using both tests.

Additional Meta-analysis Details:

Using this meta-analytical framework we were able to combine data from multiple sites and weigh individual effect size estimates by level of precision. All meta-analysis models were fit using the restricted maximum likelihood method (REML; (3)). Percent differences were calculated for each effect size difference in order to restate the difference in terms of percent change in brain volume. Percent difference is the meta-analyzed mean difference between cases and controls divided by the meta-analyzed mean volume in controls (x 100) for each trait. In addition to meta-analyzed Cohen’s *d* effect size estimates and percent differences, we calculated heterogeneity scores (I^2^) for each structure, which provide the percent of the total variance in effect size that can be explained by heterogeneity alone (4). Lower values of I^2^ indicate lower variance in the effect size estimation across studies.

References

1. Wahl I*, et al.* (2014) Standardization of depression measurement: a common metric was developed for 11 self-report depression measures. *Journal of clinical epidemiology* 67(1):73-86.

2. Puhan MA, Soesilo I, Guyatt GH, & Schunemann HJ (2006) Combining scores from different patient reported outcome measures in meta-analyses: when is it justified? *Health and quality of life outcomes* 4:94.

3. Harville DA (1977) Maximum Likelihood Approaches to Variance Component Estimation and to Related Problems. *Journal of the American Statistical Association* 72(358):320-338.

4. Higgins JP & Thompson SG (2002) Quantifying heterogeneity in a meta-analysis. *Statistics in medicine* 21(11):1539-1558.

**Supplementary Information SI2:**

We performed *post hoc* power analysis to estimate the sample sizes required to replicate the effects observed in this study. Sample size estimates are the number of subjects required in each group (in a case-control comparison) to detect an effect with 80% power at a nominal significance level (*P* = 0.05) for a two-sided t-test assuming unequal variance. All power estimates were obtained using the *pwr* package (version 1.1.1) in R.

**Supplementary Information SI3:**

**Acknowledgements:**

The **ENIGMA-Major Depressive Disorder working** group gratefully acknowledges support from the NIH BD2K award, U54EB020403.

**NESDA:** The infrastructure for the NESDA study (www.nesda.nl) is funded through the Geestkracht program of the Netherlands Organisation for Health Research and Development (Zon-Mw, grant number 10-000-1002) and is supported by participating universities (VU University Medical Center, GGZ inGeest, Arkin, Leiden University Medical Center, GGZ Rivierduinen, University Medical Center Groningen) and mental health care organizations, see [www.nesda.nl](http://www.nesda.nl/). Lianne Schmaal is supported by The Netherlands Brain Foundation Grant number F2014(1)-24.

**QTIM:** Australian National Health and Medical Research Council (Project Grants No. 496682 and 1009064 to MJ Wright and Fellowship No. 464914 to IB Hickie), US National Institute of Child Health and Human Development (RO1HD050735 to MJ Wright), and US National Institute on Drug Abuse (R00DA023549 to NA Gillespie). Baptiste Couvy-Duchesne is supported by a University of Queensland International PhD scholarship. We are grateful to the twins for their generosity of time and willingness to participate in our studies. We thank research assistants Marlene Grace, Ann Eldridge, Richard Parker, Lenore Sullivan, Lorelle Nunn, Kerrie Mcaloney, Kori Johnson, Aaron Quiggle, and Natalie Garden, radiographers Matthew Meredith, Peter Hobden, Kate Borg, Aiman Al Najjar, and Anita Burns for acquisition of the scans, and David Smyth, Anthony Conciotrorre, Daniel Park, and David Butler for IT support.

**MMDP 3T/MMDP1.5T:** Ontario Mental Health Foundation.

**Bipolar Family Study:** The Bipolar Family Study was supported by the Health Foundation and by the Dr Theresa and Mortimer Sackler Foundation.

**CODE:** The CODE cohort was collected from studies funded by Lundbeck and the German Research Foundation (WA 1539/4-1, SCHN 1205/3-1). Elizabeth Schramm is supported by the Grant of the Deutsche Forschungsgemeinschaft / German Research Association (SCHR443/11-1)

**MPIP:** The MPIP Munich Morphometry Sample comprises images acquired as part of the Munich Antidepressant Response Signature Study and the Recurrent Unipolar Depression (RUD) Case-Control study performed at the MPIP, and control subjects acquired at the Ludwig-Maximilians-University, Munich, Department of Psychiatry. We wish to acknowledge Anna Olynyik and radiographers Rosa Schirmer, Elke Schreiter, Reinhold Borschke and Ines Eidner for image acquisition and data preparation. We thank Dorothee P. Auer for local study management in the initial phase of the RUD study. The study is supported by a grant of the Exzellenz-Stiftung of the Max Planck Society. This work has also been funded by the Federal Ministry of Education and Research (BMBF) in the framework of the National Genome Research Network (NGFN), FKZ 01GS0481.

**SHIP:** The Study of Health in Pomerania (SHIP) is supported by the German Federal Ministry of Education and Research (grants 01ZZ9603, 01ZZ0103 and 01ZZ0403) the Ministry of Cultural Affairs as well as the Social Ministry of the Federal State of Mecklenburg-West Pomerania. MRI scans were supported by Siemens Healthcare, Erlangen, Germany. SHIP-LEGEND was supported by the German Research Foundation (GR1912/5-1).

**Rotterdam study:** Netherlands Organization for Scientific Research (NWO – ZonMW, VIDI grantnumber 017.106.370 to Henning Tiemeier. Dr. Meike W. Vernooij is supported by an Erasmus MC fellowship and a ZonMW clinical fellowship (90700435). We thank Daniel Bos for his contribution to this work.

**Conflicts of interest:**

All authors have no conflicts of interest related to this study. Carsten Konrad received fees for an educational program from Esparma / Aristo Pharma, Lilly, Servier, and MagVenture, as well as travel support and speakers honoraria from Lundbeck and Servier. Wiro Niessen is co-founder, chief scientific officer, and shareholder of Quantib BV. Theodorus van Erp consulted for Roche Pharmaceuticals in 2013-2014.

**Author contributions:**

Protocol design, quality testing, and meta-analysis: L.S., D.P.H., T.G.M.V., N.J.

Data collection, processing, analysis and funding: L.S., D.J.V., T.G.M.V., P.G.S., T.F., N.J.. E.L., H.T., A.H., W.J.N., M.W.V., M.A.I., K.W., H.J.G., A.B., K.H., H.V., D.H., M.C., J.L., S.N.H., I.B.H., R.G-M., B.K., O.G., B.C-D., M.E.R., L.T.S., N.T.M., G.I.D., K.L.M., S.E.M., N.G.M., N.A.G., M.J.W., G.B.H., G.M.M., E.M.F., A.C., L.S.V., M.J.V., N.J.V., I.M.V., H.W., K.S., E.S., C.N., D.S., C.K., B.Z., T.N., A.M.M., M.P., H.C.W., J.E.S., B.R.G., P.J.C., F.H.F., M.R., B.W.J.H.P., P.M.T., D.P.H.

Manuscript preparation: L.S., D.J.V., T.G.M.V., P.G.S., T.F., B.W.J.H.P., P.M.T., D.P.H.

All authors contributed edits and approved the content of the manuscript.
