# Supplementary material for: Subcortical brain alterations in major depressive disorder: findings from the ENIGMA Major Depressive Disorder working group
Source: Mol Psychiatry. 2015 Jun 30;21(6):806–12. doi: 10.1038/mp.2015.69 (PMC4879183; doi:10.1038/mp.2015.69)
Supplement: Supplementary Tables [file mp201569x2.doc]

# Supplementary Tables

**Supplementary Table S1:** ENIGMA - Major Depressive Disorder Working Group Demographics. Age (in years), sex, and MDD patients-control breakdown for participating sites

**Supplementary Table S2**: ENIGMA - Major Depressive Disorder Working Group Clinical characteristics of MDD patients. Percentage of MDD patients using antidepressant medication, percentage of first episode and recurrent episode MDD patients, percentage of acutely depressed and remitted MDD patients, severity of symptoms, and age of onset of MDD breakdown for participating sites

**Supplementary Table S3**: Instrument for diagnosing Major Depressive Disorder and exclusion criteria by site.

**Supplementary Table S4**: Image acquisition and processing by site.

**Supplementary Table S5**: Full meta-analytic results for each mean structure for the first episode MDD patients versus Controls comparison controlling for age, sex, scan center and ICV. Adjusted Cohen's d is reported.

**Supplementary Table S6**: Full meta-analytic results for each mean structure for the recurrent MDD patients versus Controls comparison controlling for age, sex, scan center and ICV. Adjusted Cohen's d is reported.

**Supplementary Table S7**: Full meta-analytic results for each mean structure for the first episode MDD patients versus recurrent MDD patients comparison controlling for age, sex, scan center and ICV. Adjusted Cohen's d is reported.

**Supplementary Table S8**: Full meta-analytic results for each mean structure for the early age of onset (≤ 21) MDD patients versus Controls comparison controlling for age, sex, scan center and ICV. Adjusted Cohen's d is reported.

**Supplementary Table S9**: Full meta-analytic results for each mean structure for the late age of onset (> 21) MDD patients versus Controls comparison controlling for age, sex, scan center and ICV. Adjusted Cohen's d is reported.

**Supplementary Table S10**: Full meta-analytic results for each mean structure for the late age of onset (> 21) MDD patients versus early age of onset (≤ 21) MDD patients comparison controlling for age, sex, scan center and ICV. Adjusted Cohen's d is reported.

**Supplementary Table S11**: Full meta-analytic results for each mean structure for the association between symptom severity and brain volumes within MDD patients based on the HDRS-17 questionnaire controlling for age, sex, scan center and ICV. Adjusted Cohen's d is reported.

**Supplementary Table S12**: Full meta-analytic results for each mean structure for the association between symptom severity and brain volumes within MDD patients based on the BDI-2 questionnaire controlling for age, sex, scan center and ICV. Adjusted Cohen's d is reported.

**Supplementary Table S13:** Full meta-analytic results for each mean structure for the MDD patients versus Controls comparison (excluding remitted patients) controlling for age, sex, scan center and ICV. Adjusted Cohen's d is reported.

**Supplementary Table 14:** Adjusted means and standard errors for each site including the total number of subjects (N) for each structure and split into MDD patients and controls (CTL). Means are adjusted for age, sex, scan center, and ICV using the *lsmeans* package in R.

**Supplementary Table 15:** Full results from the moderator analyses of mean age, field strength of scanner, percent of acute patients, FreeSurfer version used for processing, percent of patients taking antidepressants, and percent of patients taking antipsychotics. Effect sizes for the meta-regression models were available from all 15 sites (percent of patients taking antipsychotics was not available in MMDP 1.5T and MMDP 3T).

**Supplementary Table S16**: Meta-analytic results for each mean structure for the diagnosis * sex interactive effect in the full sample of MDD patients and Controls while controlling for age, sex, diagnosis, scan center and ICV. Adjusted Cohen's d is reported.

**Table S1.** ENIGMA - Major Depressive Disorder Working Group Demographics. Age (in years), sex, and MDD patients-control breakdown for participating sites

| **Study #** | **Study name** | **Age Controls (Mean ± SD)** | **Age MDD**  **(Mean ± SD)** | **% Female Controls** | **% Female MDD** | **Total N Controls** | **Total N MDD** | **Total**  **N** |
| --- | --- | --- | --- | --- | --- | --- | --- | --- |
| **1** | **NESDA** | 40.5 ± 9.8 | 37.2 ± 10.4 | 65 | 65 | 66 | 156 | 222 |
| **2** | **Imaging Genetics Dublin** | 36.7 ± 13.0 | 41.6 ± 10.8 | 56 | 63 | 52 | 52 | 104 |
| **3** | **Clinical Depression Dublin** | 30.2 ± 8.2 | 32.9 ± 9.1 | 45 | 42 | 94 | 36 | 130 |
| **4** | **CODE** | 40.0 ± 13.3 | 41.0 ± 12.0 | 58 | 65 | 74 | 102 | 176 |
| **5** | **CLING** | 25.1 ± 5.2 | 36.3 ± 11.6 | 60 | 53 | 321 | 49 | 370 |
| **6** | **SHIP** | 55.4 ± 12.8 | 53.5 ± 11.8 | 45 | 71 | 441 | 139 | 580 |
| **7** | **SHIP-trend** | 50.6 ± 14.3 | 49.4 ± 12.2 | 44 | 66 | 952 | 323 | 1275 |
| **8** | **Sydney** | 47.0 ± 23.2 | 35.8 ± 22.0 | 57 | 65 | 106 | 214 | 320 |
| **9** | **QTIM** | 22.9 ± 3.2 | 23.1 ± 2.7 | 69 | 82 | 262 | 38 | 300 |
| **10** | **Rotterdam study** | 64.6 ± 11.1 | 60.7 ± 9.8 | 53 | 72 | 4408 | 69 | 4477 |
| **11** | **Bipolar Family Study** | 23.2 ± 2.4 | 23.0 ± 3.1 | 68 | 61 | 62 | 18 | 80 |
| **12** | **DepOx** | 30.3 ± 10.0 | 30.1 ± 10.6 | 58 | 63 | 31 | 38 | 69 |
| **13** | **MPIP** | 49.1 ± 13.1 | 48.0 ± 13.8 | 58 | 56 | 222 | 368 | 590 |
| **14** | **MMDP 1.5T** | 27.4 ± 10.7 | 31.3 ± 12.2 | 64 | 51 | 44 | 53 | 97 |
| **15** | **MMDP 3T** | 30.2 ± 11.2 | 36.2 ± 14.5 | 63 | 55 | 64 | 73 | 137 |
|  | **Combined** |  |  |  |  | **7199** | **1728** | **8927** |

**Table S2.** ENIGMA - Major Depressive Disorder Working Group Clinical characteristics of MDD patients. Percentage of MDD patients using antidepressant medication, percentage of first episode and recurrent episode MDD patients, percentage of acutely depressed and remitted MDD patients, percentage of patients with a co-occurring anxiety disorder, age of onset of MDD and severity of symptoms breakdown for participating sites

| **Study #** | **Sample** | **% Antidepressants users** | **% Antipsychotics users** | **% First episode MDD/Recurrent episode MDD** | **% Acute MDD/ Remitted MDD** | **Age of onset MDD (mean ± SD)** | **% co-occurring anxiety disorder** | **HDRS-17**^a^ **Severity MDD (mean ± SD)** | **BDI-II**^b^ **Severity MDD (mean ± SD)** | **IDS-SR**^c^ **Severity MDD (mean ± SD)** |
| --- | --- | --- | --- | --- | --- | --- | --- | --- | --- | --- |
| **1** | **NESDA** | 37 | 0 | 44/56 | 100/0 | 24.3 ± 11.0 | 60 |  |  | 25.4 ± 12.0 |
| **2** | **Imaging Genetics Dublin** | 71 | 0 | 15/85 | 100/0 | 25.3 ± 12.8 | 0 | 23.6 ± 5.0 | 33.1 ± 11.7 |  |
| **3** | **Clinical Depression Dublin** | 92 | 0 | 50/50 | 100/0 | 29.6 ± 9.2 | 0 | 20.8 ± 5.1 |  |  |
| **4** | **CODE** | 0 | 0 | 0/100 | 100/0 | NA | 0 |  |  | 38.2 ± 11.6 |
| **5** | **CLING** | 94 | 12 | 45/55 | 94/6 | 30.4 ± 10.6 | 18 | 20.0 ± 4.3 | 21.9 ± 9.8 |  |
| **6** | **SHIP** | 17 | 1 | 55/45 | NA | 38.1 ± 13.2 | 37 |  | 11.7 ± 10.3 |  |
| **7** | **SHIP-trend** | 17 | 5 | 37/63 | NA | 36.2 ± 14.3 | NA |  | 12.4 ± 8.1 |  |
| **8** | **Sydney** | 61 | 24 | 12/88 | 17/83 | 24.6 ± 17.2 | 19 | 12.2 ± 6.9 |  |  |
| **9** | **QTIM** | 24 | 0 | 61/39 | 24/76 | 17.8 ± 3.5 | 29 | NA | NA |  |
| **10** | **Rotterdam study** | 29 | 4 | 45/55 | 100/0 | NA | 11 | NA | NA |  |
| **11** | **Bipolar Family Study** | 17 | 0 | NA | NA | 21.4 ± 3.3 | 0 | 5.4 ± 5.7 |  |  |
| **12** | **DepOx** | 0 | 0 | 50/50 | 100/0 | 25.6 ± 9.1 | 3 | 22.9 ± 4.3 |  |  |
| **13** | **MPIP** | 84 | 7 | 27/73 | 87/13 | 35.2 ± 14.0 | 14 | 24.9 ± 6.6 | 14.2 ± 10.9 |  |
| **14** | **MMDP 1.5T** | 25 | NA | 75/25 | 100/0 | 23.6 ± 11.5 | 19 | 12.6 ± 7.2 |  |  |
| **15** | **MMDP 3T** | 55 | NA | 45/55 | 100/0 | 23.4 ± 11.9 | 30 | 12.0 ± 7.0 |  |  |

^a^ Measured with the Hamilton Depression Rating Scale (HDRS-17; range: 0-52)

^b^ Measured with the Beck Depression Inventory (BDI-II; range: 0-63)

^c^ Measured with the Inventory of Depressive Symptomatology-Se;f report (IDS-SR; range: 0-84)

**Table S3**: Instrument for diagnosing Major Depressive Disorder and exclusion criteria by site

| **Sample** | **Instrument for diagnosing MDD** | **Exclusion criteria** |
| --- | --- | --- |
| **NESDA** | CIDI interview | MDD subjects: presence of axis-I disorders other than MDD, panic disorder, social anxiety disorder, or generalized anxiety disorder and any use of psychotropic medication other than stable use of SSRIs or infrequent benzodiazepine use (i.e., equivalent to 2 doses of 10 mg of oxazepam 3 times per week or use within 48 hours prior to scanning).  Control subjects: no Axis-I diagnosis, no medication use.  All subjects: presence or history of major internal or neurological disorder, dependence on or recent abuse (past year) of alcohol and/or drugs, hypertension, and general MRI contraindications. |
| **Imaging Genetics Dublin** | SCID-1 interview | MDD subjects: comorbid psychiatric disorders (Axis I or Axis II, other than MDD), Treatment with antipsychotics or mood stabilizers, age <18 or >65,  Control subjects: no Axis-I diagnosis, no medication use.  All subjects: history of neurological or other severe medical illness, head injury or severe substance abuse in their lifetime history and general MRI contraindications. |
| **Clinical Depression Dublin** | SCID-1 interview | MDD subjects: comorbid psychiatric disorders (Axis I or Axis II, other than MDD), Treatment with antipsychotics or mood stabilizers, age <18 or >65,  Control subjects: no Axis-I diagnosis, no medication use.  All subjects: history of neurological or other severe medical illness, head injury or severe substance abuse in their lifetime history and general MRI contraindications. |
| **CODE** | SCID interview | MDD: Presence of any other Axis-1 diagnosis; Acute risk for suicide (in contrast to suicidal ideation); History of psychotic symptoms, bipolar disorder, or dementia; Schizotypal, antisocial or borderline personality disorder; Use of psychotropic medication within two weeks prior to the start of the study; No current psychotherapeutic treatment.  Control subjects: No history of or current Axis-1 or 2 disorders.  All subjects: History of or current neurological disorder or brain injury; Serious medical condition; Severe cognitive impairment; Substance-related abuse or dependence disorder; Use of psychotropic medication; Use of central-acting medication; Pregnancy; General MRI contraindications. |
| **CLING** | ICD-10 interview | MDD subjects: past or actual presence of other axis I diagnoses other than anxiety disorders, alcohol/cannabis abuse and tobacco dependence; neurological or other medical conditions that could be related to affective symptoms  Control subjects: no medical history, including neurological and psychiatric history, as well as no previous or actual use of psychotropic medication |
| **SHIP** | M-CIDI interview | MDD subjects: presence of axis-I disorders other than MDD, anxiety disorders, conversion, somatization and eating disorder.  Control subjects: no lifetime diagnosis of depression, no antidepressiva, and severity index=0  All subjects: We removed subjects with medical conditions (e.g. a history of cerebral tumor, stroke, Parkinson’s diseases, multiple sclerosis, epilepsy, hydrocephalus, enlarged ventricles, pathological lesions) or due to technical reasons (e.g. severe movement artifacts or inhomogeneity of the magnetic field). |
| **SHIP-trend** | M-CIDI interview | MDD subjects: no special exclusion criteria  Control subjects: no lifetime diagnosis of depression, no antidepressiva, and severity index=0  All subjects: We removed subjects with due to medical conditions (e.g. a history of cerebral tumor, stroke, Parkinson’s diseases, multiple sclerosis, epilepsy, hydrocephalus, enlarged ventricles, pathological lesions) or due to technical reasons (e.g. severe movement artifacts or inhomogeneity of the magnetic field). |
| **Sydney** | SCID interview | MDD subjects: presence of axis-I disorders other than MDD, panic disorder, social anxiety disorder, or generalized anxiety disorder.  Control subjects: no Axis-I diagnosis, no medication use.  Exclusion criteria for all subjects included medical instability (as determined by a psychiatrist), history of neurological disease (e.g. tumour, head trauma, epilepsy), medical illness known to impact cognitive and brain function (e.g. cancer), intellectual and/or developmental disability and insufficient English for neuropsychological assessment. All subjects were asked to abstain from drug or alcohol use for 48 hours prior to testing and informed about a drug screen protocol. |
| **QTIM** | CIDI interview | MDD subjects: presence of axis-I disorders other than MDD, anxiety disorders  Control subjects: antidepressant use, psychiatric disorders  All subjects: relatedness between subjects, left handedness, history of neurological or other severe medical illness, head injury or current or past diagnosis of substance abuse, use of cognition affecting medication and general MRI contraindications |
| **Rotterdam study** | SCAN interview | MDD subjects: Persons who screened positive for depressive symptoms on CESD but did not meet criteria for MDD from SCAN interview. Persons who screened positive for depressive symptoms on CESD and then did not undergo SCAN interview. Presence of axis-I disorders other than MDD and anxiety disorders (DSM-IV). Persons with MRI contraindications.  Control subjects: use of psychoanaleptics, MRI contraindications. |
| **Bipolar Family Study** | SCID interview | MDD subjects: presence of other axis I diagnoses.  Control subjects: no medical history, including neurological and psychiatric history, as well as no previous or actual use of psychotropic medication  All subjects: any major neurological disorder, learning disability, or any history of head injury that included loss of consciousness and any contraindications to MRI. |
| **DepOx** | SCID interview | MDD subjects: presence of axis-I disorders other than MDD and anxiety disorders (DSM-IV), clinically significant risk of suicidal behaviour, having contraindications to escitalopram treatment or being treated with psychotropic medication less than three weeks before the study (five weeks in the case of fluoxetine)  Control subjects: current or past history of Axis I disorder as defined by DSM-IV  Both groups: major somatic or neurological disorders, pregnancy or breast-feeding, contra-indications to MR imaging or concurrent medication which could alter emotional processing |
| **MPIP** | M-CIDI/SCAN interview | 1. Munich Antidepressant Response Signature (MARS) study  MDD subjects (clinical consensus diagnosis or M-CIDI (since 2008)): depressive syndromes secondary to any medical or neurological condition (e. g., in­toxi­cation, drug abuse, stroke), the presence of manic, hypomanic or mixed aﬀective symptoms, lifetime diagnosis of alcohol dependence, illicit drug abuse or the presence of severe medical conditions (e.g., ischemic heart disease). Patients with bipolar depression were excluded for the current MR study.  Control subjects: age > 65, MMSE<27, presence of severe somatic diseases or lifetime history of the following axis I disorders as assessed by the M-CIDI interview: alcohol dependence, drug abuse or dependence, possible psychotic disorder, mood disorder, anxiety disorder including OCD and PTSD, somatoform disorder, dissociative disorder NOS, and eating disorder  2. Recurrent unipolar depression (RUD) study  MDD subjects (SCAN interview): presence of manic episodes, mood incongruent psychotic symptoms, the presence of a lifetime diagnosis of intravenous drug abuse and depressive symptoms only secondary to alcohol or substance abuse or to medical illness or medication.  Control subjects: presence of severe somatic diseases or life-time history of anxiety and affective disorders according to the Composite International Diagnostic-Screener (CIDI-S)  All subjects: gross incidental MR findings such as territorial infarction, tumor, hydrocephalus, malformations and anatomical deviations (e.g. enlarged ventricles) that prevent appropriate image processing were additional exclusion criteria.  3. MR images of 9 additional controls acquired at the LMU, Munich, meeting equivalent criteria as the RUD control sample were included. |
| **MMDP 3T** | SCID interview | MDD subjects: presence of axis-I disorders other than MDD and anxiety disorders (DSM-IV).  Control subjects had to have no psychiatric history, as assessed with the SCID-I/P  Symptom severity was assessed using the 17-item Hamilton Depression Rating Scale (HDRS-17) and the Global Assessment of Functioning Scale (GAF). Control subjects also received these measures to rule out the presence of sub-threshold psychiatric illness.  Exclusion criteria for all groups were:  (1) substance-use related disorder within the past 6 months as determined by the SCID;  (2) lifetime history of substance dependence as measured by the SCID;  (3) PTSD as determined by the SCID;  (4) treatment with anti-cholinergic or typical (first generation) anti-psychotic medication;  (5) use of alcohol or illicit psychoactive substance within 48 h of testing;  (6) untreated medical illness such as uncontrolled diabetes or other endocrine disorders eg. Cushing’s;  (7) history of head injury with loss of consciousness;  (8) history of neurological disease; and  (9) past treatment with electroconvulsive therapy (ECT), transcranial magnetic stimulation, or psychotherapy within the past year.  (10) English comprehension lower than a grade 6 reading level. |
| **MMDP 1.5T** | SCID interview | MDD subjects: presence of axis-I disorders other than MDD and anxiety disorders (DSM-IV),  Control subjects had to have no psychiatric history, as assessed with the SCID-I/P  Symptom severity was assessed using the 17-item Hamilton Depression Rating Scale (HDRS-17) and the Global Assessment of Functioning Scale (GAF). Control also received these measures to rule out the presence of sub-threshold psychiatric illness.  Exclusion criteria for all groups were:  (1) substance-use related disorder within the past 6 months as determined by the SCID;  (2) lifetime history of substance dependence as measured by the SCID;  (3) PTSD as determined by the SCID;  (4) treatment with anti-cholinergic or typical (first generation) anti-psychotic medication;  (5) use of alcohol or illicit psychoactive substance within 48 h of testing;  (6) untreated medical illness such as uncontrolled diabetes or other endocrine disorders eg. Cushing’s;  (7) history of head injury with loss of consciousness;  (8) history of neurological disease; and  (9) past treatment with electroconvulsive therapy (ECT), transcranial magnetic stimulation, or psychotherapy within the past year.  (10) English comprehension lower than a grade 6 reading level. |

MDD: Major Depressive Disorder; CIDI: the Composite International Diagnostic Interview; SCID: Structured Clinical Interview for DSM disorders; SCAN: Schedules for Clinical Assessment in Neuropsychiatry; CESD: Center for Epidemiologic Studies Depression scale; DSM: Diagnostic and Statistical Manual of Mental Disorders; MRI: Magnetic Resonance Imaging; OCD: Obsessive Compulsive Disorder; PTSD: Posttraumatic Stress Disorder.

**Table S4**: Image acquisition and processing by site

| **Sample** | **Scanner vendor and type** | **Acquisition parameters** | **Freesurfer version** | **Slice orientation** | **Operating system** |
| --- | --- | --- | --- | --- | --- |
| **NESDA** | 3T Phillips Achieva/Intera | 3D gradient-echo T1-weighted sequence. TR=9 msec; TE=3.5 msec; flip angle 8º, FOV = 256 mm; matrix: 25x62x56; in plane voxel size = 1 mm × 1 mm x 1 mm; 170 slices. | 5.0 | Sagittal | Linux-centos4_x86_64 |
| **Imaging Genetics Dublin** | 3T Phillips Achieva | A sagittal T1 3D TFE was used to scan all participants. TR=8.5 msec; TE=3.9 msec; FOV = 256 mm, AP: 256 mm, RL: 160 mm; matrix: 256×256. | 5.3 | Sagittal | Mac OS |
| **Clinical Depression Dublin** | 1.5T Siemens Vision | 3D-MPRAGE T1-weighted sequence. TR=11.6 msec; TE=4.9 msec; FOV=230 mm; matrix 512 x 512, slice thickness: 1.5 mm. | 5.3 | Coronal | Mac OS |
| **CODE** | 3T Siemens Trio (4 Sites), 3 T Philips Achieva (1 site) | Siemens: T1 mprage, voxel size 1 mm x 1 mm x 1 mm; TR=1900 msec; TE=2.52 msec; Sample 1: 192 slices, Sample 2: 176 slices (except 1 site: 192)  Philips: T1 3D-TFE, voxel size 1 mm x 1 mm x 1 mm; TR=8.3 msec; TE=3.8 msec; 170 slices. | 5.3 | Sagittal | Ubuntu 12.04 LTS (Linux 64bit) |
| **CLING** | 3T Siemens Tim Trio | Standard 3D T1-weighted turbo fast low angle shot (turbo FLASH); voxel size 1 mm x 1 mm x 1mm (based on the ADNI protocol (Jack et al. 2008); TR=225 msec; TE=3.26 msec, FOV=256 x 256 x 192 | 5.1 | Sagittal | Linux |
| **SHIP** | 1.5T Siemens Avanto | 3D T1-weighted (MP-RAGE/ axial plane); TR=1900 msec; TE=3.4 msec; Flip angle=15°; voxel size 1 mm x 1 mm x 1 mm | 5.1 | Axial | Centos6_x86_64 |
| **SHIP-trend** | 1.5T Siemens Avanto | 3D T1-weighted (MP-RAGE/ axial plane); TR=1900 msec; TE=3.4 msec; Flip angle=15°; voxel size 1 mm x 1 mm x 1 mm | 5.1 | Axial | Centos6_x86_64 |
| **Sydney** | 3T GE MR750 | 3D T1-weighted sequence. TR=7.2 msec; TE=2.78 msec; matrix =256; FOV=240; No. slices=196; thick=0.9mm; inplane resolution=0.9375 | 5.1 | Coronal | Linux_Ubuntu12.04_64 |
| **QTIM** | Bruker 4T Whole-body MRI | 3D T1 weighted sequence. TR=1500 msec; TE=3.35 msec; flip angle=8°, 256 or 240 (coronal or sagittal) slices, FOV=240 mm, matrix 256x256x256 (or 256x256x240) | 5.1 | Coronal, then sagittal following software upgrade. | Linux-centos4_x86_64-stable-pub-v5.1.0 |
| **Rotterdam study** | 1.5T Signa Excite -General Electric Healthcare,  Milwaukee, USA, software version 11x | 3D GRE T1 weighted sequence. TR= 13.8 msec; TE=2.8 msec; TI=400 ms; Flip angle=20°; FOV=25cm2; maxtrix 416 x 256; voxel size 1 mm x 1 mm x 1 mm. | 4.5 | Axial |  |
| **Bipolar Family Study** | 1.5T GE Signa | T1-weighted sequence. TR=500 msec; TE=4 msec; flip angle 8°; matrix 192 x 192; 180 slices; voxel size 1.25 mm x 1.25 mm x 1.20 mm; FOV=24, phase FOV 1 | 5.3 | Coronal | linux 6, x86_64, kernel 2.6.32 |
| **DepOx** | 3T Siemens Tim Trio | T1 weighted sequence. TR=1100 msec; TE=4.8 msec; TI=2040 msec; voxel size 0.78 mm x 0.8 mm x 0.78 mm on a 208 x 256 x 200 grid, | 5.3 | Transversal | Suse Linux x86_64 |
| **MPIP** | 1.5T GE and Siemens (the latter: only few cases) | #1: T1-weighted SPGR sagittal 3D volume. TR=1030 msec; TE=3.4 msec; 124 slices; matrix=256x256; FOV=23.0x23.0 cm2; voxel size=0.8975 mm x0.8975 mm x 1.2-1.4 mm; flip angle=90°; birdcage resonator.  #2: same scanner as #1, platform update Signa Excite, sagittal T1-weighted (spin echo sequence, TR=9.7 msec, TE=2.1 msec; FOV=25.0x25.0 cm2, voxel size=0.875 mm x0.875 mm x1.2 mm, 124-132 slices, flip angle=90°.    #3: Siemens 1.5 Tesla, Vario, 3D MPRAGE, TR=11.6 msec; TE=4.9 msec; FOV 23x23 cm2; matrix 512x512; 126 axial slices; voxel site 0.45 mm x 0.45 mm x 1.5 mm. (only N=2 subjects) | 5.3 | 1.5 GE: sagittal  1.5 Siemens: axial | Linux 2.6.37.1-1.2-desktop x86_64 |
| **MMDP 3T** | 3T MRI Signa GE Excite | Axial T-1 weighted sequence; 3D SPGR pulse; fast IRP sequence; TR = 7.012 msec; TE = 2.1 msec; Ti=450 msec; flip angle= 12°; FOV = 240; slice thickness = 2 mm no skip; frequency matrix = 320; phase matrix = 192; frequency direction = A/P | 5.3 | Axial |  |
| **MMDP 1.5T** | 1.5-T. Signa GE Genesis-based Echo-Speed scanner | Sagittal anatomic images were acquired by using a 3D/FSPGR/20 sequence. flip angle=20; TR=300 msec; inversion recovery=300 msec; matrix=512x256; FOV=24 cm; scan thickness=1.2 mm | 5.3 | Sagittal |  |

3D: three-dimensional; TR: repetition time; TE: echo time; FOV: field of view

**Supplementary Table S5**: Full meta-analytic results for each mean structure for the first episode MDD patients versus Controls comparison controlling for age, sex, scan center and ICV. Adjusted Cohen's d is reported.

|  | **Cohen's d ^a^**  **(First episode MDD - CTL)** | **Std. Err.** | **95% CI** | **% Difference** | **P-value** | **I^2^** | **# Controls** | **# Patients** |
| --- | --- | --- | --- | --- | --- | --- | --- | --- |
| **Lateral Ventricles** | 0.034 | 0.049 | [-0.061 - 0.129] | 0.601 | 0.485 | <0.001 | 6922 | 567 |
| **Thalamus** | -0.047 | 0.049 | [-0.142 - 0.049] | -0.575 | 0.339 | <0.001 | 6910 | 566 |
| **Caudate** | -0.009 | 0.088 | [-0.181 - 0.164] | -0.542 | 0.923 | 65.118 | 6898 | 573 |
| **Putamen** | -0.032 | 0.061 | [-0.151 - 0.088] | -0.652 | 0.605 | 27.697 | 6821 | 557 |
| **Pallidum** | -0.008 | 0.049 | [-0.104 - 0.089] | -0.286 | 0.876 | <0.001 | 6882 | 558 |
| **Hippocampus** | -0.073 | 0.074 | [-0.219 - 0.073] | -0.762 | 0.325 | 51.155 | 6904 | 572 |
| **Amygdala** | -0.041 | 0.057 | [-0.153 - 0.072] | -0.317 | 0.478 | 20.999 | 6924 | 570 |
| **Accumbens** | 0.012 | 0.066 | [-0.117 - 0.142] | -0.117 | 0.853 | 37.094 | 6831 | 550 |
| **ICV** | -0.005 | 0.055 | [-0.113 - 0.103] | -0.071 | 0.930 | 17.697 | 7063 | 583 |

**^a^** Included Samples: NESDA, Clinical Depression Dublin, CLING, SHIP, SHIP-trend, Sydney, QTIM, Rotterdam study, DepOx, MPIP, MMDP 3T, MMDP 1.5T.

ICV: Intracranial Volume; MDD: Major Depressive Disorder; CTL: Controls.

**Supplementary Table S6**: Full meta-analytic results for each mean structure for the recurrent MDD patients versus Controls comparison controlling for age, sex, scan center and ICV. Adjusted Cohen's d is reported.

|  | **Cohen's d ^a^**  **(Recurrent MDD - CTL)** | **Std. Err.** | **95% CI** | **% Difference** | **P-value** | **I^2^** | **# Controls** | **# Patients** |
| --- | --- | --- | --- | --- | --- | --- | --- | --- |
| **Lateral Ventricles** | 0.067 | 0.040 | [-0.01 - 0.145] | 2.096 | 0.089 | <0.001 | 6996 | 1096 |
| **Thalamus** | 0.001 | 0.040 | [-0.078 - 0.079] | -0.042 | 0.990 | <0.001 | 6984 | 1090 |
| **Caudate** | 0.015 | 0.052 | [-0.086 - 0.117] | 0.268 | 0.768 | 29.644 | 6972 | 1082 |
| **Putamen** | 0.045 | 0.043 | [-0.039 - 0.128] | 0.404 | 0.294 | 5.850 | 6895 | 1073 |
| **Pallidum** | 0.010 | 0.040 | [-0.069 - 0.089] | 0.114 | 0.799 | <0.001 | 6956 | 1073 |
| **Hippocampus** | -0.174 | 0.040 | [-0.252 - -0.096] | -1.443 | 1.12E-05 | <0.001 | 6978 | 1102 |
| **Amygdala** | -0.070 | 0.040 | [-0.148 - 0.008] | -0.814 | 0.077 | <0.001 | 6998 | 1100 |
| **Accumbens** | -0.043 | 0.040 | [-0.121 - 0.036] | -0.612 | 0.288 | <0.001 | 6905 | 1076 |
| **ICV** | 0.017 | 0.046 | [-0.074 - 0.108] | 0.135 | 0.712 | 18.621 | 7137 | 1119 |

**^a^** Included Samples: NESDA, Imaging Genetics Dublin, Clinical Depression Dublin, CODE, CLING, SHIP, SHIP-trend, Sydney, QTIM, Rotterdam study, DepOx, MPIP, MMDP 3T, MMDP 1.5T.

ICV: Intracranial Volume; MDD: Major Depressive Disorder; CTL: Controls.

**Supplementary Table S7**: Full meta-analytic results for each mean structure for the recurrent MDD patients versus first episode MDD patients comparison controlling for age, sex, scan center and ICV. Adjusted Cohen's d is reported.

|  | **Cohen's d ^a^ (Recurrent MDD - First episode MDD)** | **Std. Err.** | **95% CI** | **% Difference** | **P-value** | **I^2^** | **# First MDD** | **# Recur MDD** |
| --- | --- | --- | --- | --- | --- | --- | --- | --- |
| **Lateral Ventricles** | 0.015 | 0.056 | [-0.095 - 0.124] | 1.262 | 0.795 | <0.001 | 567 | 994 |
| **Thalamus** | 0.053 | 0.056 | [-0.056 - 0.163] | 0.488 | 0.341 | <0.001 | 566 | 988 |
| **Caudate** | -0.020 | 0.056 | [-0.13 - 0.09] | -0.049 | 0.718 | <0.001 | 573 | 980 |
| **Putamen** | 0.033 | 0.090 | [-0.144 - 0.21] | 0.980 | 0.715 | 52.590 | 557 | 971 |
| **Pallidum** | -0.015 | 0.058 | [-0.128 - 0.098] | 0.026 | 0.795 | 1.968 | 558 | 971 |
| **Hippocampus** | -0.069 | 0.079 | [-0.223 - 0.085] | -0.701 | 0.379 | 39.712 | 572 | 1000 |
| **Amygdala** | -0.035 | 0.056 | [-0.145 - 0.075] | -0.528 | 0.531 | <0.001 | 570 | 999 |
| **Accumbens** | -0.093 | 0.066 | [-0.222 - 0.037] | -0.865 | 0.160 | 17.638 | 550 | 975 |
| **ICV** | 0.030 | 0.055 | [-0.079 - 0.138] | 0.362 | 0.593 | <0.001 | 583 | 1017 |

**^a^** Included Samples: NESDA, Clinical Depression Dublin, CLING, SHIP, SHIP-trend, Sydney, QTIM, Rotterdam study, DepOx, MPIP, MMDP 3T, MMDP 1.5T.

% Difference is difference between recurrent MDD patients and first episode MDD patients divided by the average volume in first episode MDD patients.

ICV: Intracranial Volume; MDD: Major Depressive Disorder; CTL: Controls.

**Supplementary Table S8**: Full meta-analytic results for each mean structure for the early age of onset (≤ 21) MDD patients versus Controls comparison controlling for age, sex, scan center and ICV. Adjusted Cohen's d is reported.

|  | **Cohen's d ^a^ (EAO MDD - CTL)** | **Std. Err.** | **95% CI** | **% Difference** | **P-value** | **I^2^** | **# Controls** | **# Patients** |
| --- | --- | --- | --- | --- | --- | --- | --- | --- |
| **Lateral Ventricles** | 0.145 | 0.055 | [0.037 - 0.253] | 5.107 | 8.50E-03 | <0.001 | 2628 | 533 |
| **Thalamus** | -0.067 | 0.055 | [-0.175 - 0.042] | -0.678 | 0.228 | 0.003 | 2603 | 531 |
| **Caudate** | 0.020 | 0.073 | [-0.123 - 0.163] | 0.093 | 0.783 | 36.502 | 2597 | 529 |
| **Putamen** | 0.020 | 0.067 | [-0.111 - 0.151] | 0.075 | 0.763 | 24.492 | 2533 | 525 |
| **Pallidum** | 0.047 | 0.060 | [-0.071 - 0.165] | 0.538 | 0.434 | 10.892 | 2572 | 526 |
| **Hippocampus** | -0.205 | 0.056 | [-0.314 - -0.096] | -1.846 | 2.31E-04 | 0.579 | 2603 | 533 |
| **Amygdala** | -0.117 | 0.055 | [-0.225 - -0.01] | -1.228 | 0.033 | <0.001 | 2612 | 537 |
| **Accumbens** | -0.058 | 0.056 | [-0.167 - 0.052] | -0.830 | 0.302 | <0.001 | 2504 | 526 |
| **ICV** | 0.041 | 0.080 | [-0.116 - 0.199] | -0.173 | 0.607 | 48.751 | 2717 | 541 |

**^a^** Included Samples: NESDA, Imaging Genetics Dublin, CLING, SHIP, SHIP-trend, Sydney, QTIM, DepOx, MPIP, MMDP 3T, MMDP 1.5T.

ICV: Intracranial Volume; EAO: Early Age of Onset; MDD: Major Depressive Disorder; CTL: Controls.

**Supplementary Table S9**: Full meta-analytic results for each mean structure for the late age of onset (> 21) MDD patients versus Controls comparison controlling for age, sex, scan center and ICV. Adjusted Cohen's d is reported.

|  | **Cohen's d ^a^ (LAO MDD - CTL)** | **Std. Err.** | **95% CI** | **% Difference** | **P-value** | **I^2^** | **# Controls** | **# Patients** |
| --- | --- | --- | --- | --- | --- | --- | --- | --- |
| **Lateral Ventricles** | -0.005 | 0.041 | [-0.085 - 0.075] | -0.314 | 0.911 | <0.001 | 2628 | 967 |
| **Thalamus** | 0.034 | 0.044 | [-0.053 - 0.12] | 0.262 | 0.447 | 7.019 | 2603 | 962 |
| **Caudate** | -0.051 | 0.052 | [-0.153 - 0.052] | -0.683 | 0.332 | 24.946 | 2597 | 965 |
| **Putamen** | 0.013 | 0.045 | [-0.074 - 0.101] | 0.039 | 0.766 | 7.080 | 2533 | 943 |
| **Pallidum** | -0.032 | 0.041 | [-0.113 - 0.05] | -0.373 | 0.446 | 0.009 | 2572 | 942 |
| **Hippocampus** | -0.103 | 0.053 | [-0.207 - 0.001] | -0.818 | 0.052 | 27.690 | 2603 | 978 |
| **Amygdala** | -0.028 | 0.048 | [-0.123 - 0.067] | -0.343 | 0.568 | 17.350 | 2612 | 971 |
| **Accumbens** | -0.030 | 0.042 | [-0.112 - 0.052] | -0.278 | 0.471 | <0.001 | 2504 | 938 |
| **ICV** | 0.003 | 0.074 | [-0.142 - 0.148] | 0.213 | 0.968 | 61.068 | 2717 | 997 |

**^a^** Included Samples: NESDA, Imaging Genetics Dublin, Clinical Depression Dublin, CLING, SHIP, SHIP-trend, Sydney, DepOx, MPIP, MMDP 3T, MMDP 1.5T.

ICV: Intracranial Volume; MDD: LAO: Late Age of Onset; Major Depressive Disorder; CTL: Controls.

**Supplementary Table S10**: Full meta-analytic results for each mean structure for the late age of onset (> 21) MDD patients versus early age of onset (≤ 21) MDD patients comparison controlling for age, sex, scan center and ICV. Adjusted Cohen's d is reported.

|  | **Cohen's d ^a^ (LAO MDD – EAO MDD)** | **Std. Err.** | **95% CI** | **% Difference** | **P-value** | **I^2^** | **# Controls** | **# Patients** |
| --- | --- | --- | --- | --- | --- | --- | --- | --- |
| **Lateral Ventricles** | -0.133 | 0.062 | [-0.254 - -0.011] | -7.101 | 0.032 | <0.001 | 533 | 967 |
| **Thalamus** | 0.096 | 0.070 | [-0.042 - 0.234] | 1.554 | 0.173 | 15.429 | 531 | 962 |
| **Caudate** | -0.059 | 0.077 | [-0.21 - 0.092] | -1.209 | 0.442 | 26.757 | 529 | 965 |
| **Putamen** | -0.028 | 0.070 | [-0.164 - 0.109] | -0.625 | 0.691 | 13.196 | 525 | 943 |
| **Pallidum** | -0.082 | 0.090 | [-0.258 - 0.094] | -2.064 | 0.359 | 43.465 | 526 | 942 |
| **Hippocampus** | 0.143 | 0.066 | [0.013 - 0.272] | 2.228 | 0.031 | 8.355 | 533 | 978 |
| **Amygdala** | 0.120 | 0.065 | [-0.008 - 0.247] | 1.940 | 0.066 | 5.954 | 537 | 971 |
| **Accumbens** | 0.074 | 0.063 | [-0.049 - 0.198] | 1.079 | 0.238 | <0.001 | 526 | 938 |
| **ICV** | -0.074 | 0.096 | [-0.262 - 0.114] | -0.609 | 0.439 | 52.258 | 541 | 997 |

**^a^** Included Samples: NESDA, Imaging Genetics Dublin, CLING, SHIP, SHIP-trend, Sydney, DepOx, MPIP, MMDP 3T, MMDP 1.5T.

% Difference is difference between late age of onset (> 21) MDD patients and early age of onset (≤ 21) MDD patients divided by the average volume in late age of onset (> 21) MDD patients.

ICV: Intracranial Volume; LAO: Late Age of Onset; EAO: Early Age of Onset; MDD: Major Depressive Disorder.

**Supplementary Table S11**: Full meta-analytic results for each mean structure for the association between symptom severity and brain volumes within MDD patients based on the HDRS-17 questionnaire controlling for age, sex, scan center and ICV. Adjusted Cohen's d is reported.

|  | **Pearson's r ^a^** | **Std. Err.** | **95% CI** | **P-value** | **I^2^** | **# Patients** |
| --- | --- | --- | --- | --- | --- | --- |
| **Lateral Ventricles** | -0.013 | 0.066 | [-0.142 - 0.117] | 0.847 | 59.501 | 659 |
| **Thalamus** | 0.018 | 0.039 | [-0.058 - 0.094] | 0.640 | 0.003 | 658 |
| **Caudate** | 0.076 | 0.039 | [-0.001 - 0.152] | 0.053 | <0.001 | 654 |
| **Putamen** | 0.005 | 0.039 | [-0.072 - 0.081] | 0.900 | <0.001 | 657 |
| **Pallidum** | -0.018 | 0.039 | [-0.095 - 0.058] | 0.639 | <0.001 | 656 |
| **Hippocampus** | 0.109 | 0.068 | [-0.024 - 0.241] | 0.109 | 62.572 | 664 |
| **Amygdala** | 0.022 | 0.050 | [-0.075 - 0.119] | 0.659 | 29.854 | 667 |
| **Accumbens** | 0.017 | 0.039 | [-0.059 - 0.093] | 0.663 | 0.020 | 658 |
| **ICV** | 0.002 | 0.039 | [-0.073 - 0.078] | 0.956 | <0.001 | 667 |

**^a^** Included samples: Imaging Genetics Dublin, Clinical Depression Dublin, CLING, Sydney, Bipolar Family Study, DepOx, MPIP, MMDP 3T, MMDP 1.5T.

HDRS-17: Hamilton Rating Scale for Depression 17 items; ICV: Intracranial Volume; MDD: Major Depressive Disorder.

**Supplementary Table S12**: Full meta-analytic results for each mean structure for the association between symptom severity and brain volumes within MDD patients based on the BDI-2 questionnaire controlling for age, sex, scan center and ICV. Adjusted Cohen's d is reported.

|  | **Pearson's r ^a^** | **Std. Err.** | **95% CI** | **P-value** | **I^2^** | **# Patients** |
| --- | --- | --- | --- | --- | --- | --- |
| **Lateral Ventricles** | -0.020 | 0.076 | [-0.168 - 0.128] | 0.791 | 66.343 | 634 |
| **Thalamus** | -0.048 | 0.056 | [-0.157 - 0.062] | 0.391 | 38.219 | 624 |
| **Caudate** | -0.062 | 0.040 | [-0.14 - 0.016] | 0.120 | <0.001 | 628 |
| **Putamen** | -0.034 | 0.041 | [-0.114 - 0.046] | 0.408 | <0.001 | 603 |
| **Pallidum** | 0.028 | 0.041 | [-0.053 - 0.108] | 0.500 | <0.001 | 599 |
| **Hippocampus** | -0.058 | 0.053 | [-0.161 - 0.045] | 0.271 | 34.161 | 646 |
| **Amygdala** | -0.016 | 0.040 | [-0.094 - 0.061] | 0.677 | 0.059 | 639 |
| **Accumbens** | -0.061 | 0.041 | [-0.141 - 0.018] | 0.130 | 0.008 | 599 |
| **ICV** | -0.080 | 0.039 | [-0.156 - -0.005] | 0.037 | 0.496 | 667 |

**^a^** Included samples: Imaging Genetics Dublin, CLING, SHIP, SHIP-trend, MPIP.

BDI-2: Beck Depression Inventory second edition; ICV: Intracranial Volume; MDD: Major Depressive Disorder.

**Supplementary Table S13:** Full meta-analytic results for each mean structure for the MDD patients versus Controls comparison (excluding remitted patients) controlling for age, sex, scan center and ICV. Adjusted Cohen's d is reported.

|  | **Cohen's d ^a^ (MDD - CTL)** | **Std. Err.** | **95% CI** | **% Difference** | **P-value** | **I^2^** | **# Controls** | **# Patients** |
| --- | --- | --- | --- | --- | --- | --- | --- | --- |
| **Lateral Ventricles** | 0.081 | 0.060 | [-0.036 - 0.198] | 1.179 | 0.175 | <0.001 | 4779 | 570 |
| **Thalamus** | -0.108 | 0.075 | [-0.256 - 0.04] | -1.040 | 0.152 | 33.014 | 4780 | 569 |
| **Caudate** | -0.015 | 0.061 | [-0.135 - 0.106] | -0.258 | 0.808 | 3.435 | 4782 | 563 |
| **Putamen** | 0.022 | 0.066 | [-0.108 - 0.153] | 0.146 | 0.737 | 15.933 | 4766 | 568 |
| **Pallidum** | -0.011 | 0.060 | [-0.128 - 0.107] | -0.174 | 0.855 | <0.001 | 4788 | 567 |
| **Hippocampus** | -0.189 | 0.060 | [-0.306 - -0.072] | -1.534 | 0.002 | <0.001 | 4787 | 575 |
| **Amygdala** | -0.036 | 0.064 | [-0.162 - 0.09] | -0.373 | 0.577 | 12.667 | 4798 | 576 |
| **Accumbens** | -0.034 | 0.060 | [-0.151 - 0.083] | -0.538 | 0.569 | <0.001 | 4812 | 572 |
| **ICV** | -0.046 | 0.061 | [-0.165 - 0.073] | -0.446 | 0.449 | 3.571 | 4833 | 579 |

^a^ Included samples: NESDA, Imaging Genetics Dublin, Clinical Depression Dublin, CODE, Rotterdam study, DepOx, MMDP 1.5T and MMDP 3T

ICV: Intracranial Volume; MDD: Major Depressive Disorder; CTL: Controls.

**Supplementary Table 14:** Adjusted means and standard errors for each site including the total number of subjects (N) for each structure and split into MDD patients and controls (CTL). Means are adjusted for age, sex, scan center, and ICV using the *lsmeans* package in R.

|  |  | **Lateral Ventricles** | | **Thalamus** | | **Caudate** | | **Putamen** | | **Pallidum** | | **Hippocampus** | | **Amygdala** | | **Accumbens** | | **ICV** | |
| --- | --- | --- | --- | --- | --- | --- | --- | --- | --- | --- | --- | --- | --- | --- | --- | --- | --- | --- | --- |
|  |  | Mean  (Std Err) | N | Mean  (Std Err) | N | Mean (Std Err) | N | Mean  (Std Err) | N | Mean (Std Err) | N | Mean (Std Err) | N | Mean (Std Err) | N | Mean (Std Err) | N | Mean  (Std Err) | N |
| **Bipolar Family Study** | CTL | 5889.994 (310.279) | 62 | 6598.157 (61.08) | 62 | 3761.069 (54.768) | 62 | 5891.301 (76.594) | 62 | 1893.376 (27.259) | 62 | 3570.397 (42.926) | 62 | 1878.994 (28.872) | 62 | 709.989 (17.126) | 62 | 1478595.321 (15147.359) | 62 |
|  | MDD | 5564.152 (565.765) | 19 | 6407.857 (111.373) | 19 | 3549.248 (99.865) | 19 | 5635.256 (139.662) | 19 | 1905.38 (49.703) | 19 | 3454.205 (78.272) | 19 | 1826.493 (52.646) | 19 | 742.377 (31.227) | 19 | 1429565.795 (27391.122) | 19 |
| **CLING** | CTL | 6942.48 (211.514) | 321 | 8169.268 (36.942) | 321 | 4038.22 (23.439) | 321 | 5694.382 (30.049) | 321 | 1830.209 (10.654) | 321 | 4320.772 (18.88) | 321 | 1511.964 (7.769) | 321 | 600.179 (4.461) | 321 | 1591240.917 (8643.52) | 321 |
|  | MDD | 7237.454 (608.396) | 49 | 8076.854 (106.259) | 49 | 4037.733 (67.419) | 49 | 5744.305 (86.431) | 49 | 1828.344 (30.646) | 49 | 4228.621 (54.308) | 49 | 1545.63 (22.348) | 49 | 585.005 (12.832) | 49 | 1528713.378 (24731.27) | 49 |
| **Clinical Depression Dublin** | CTL | 6988.118 (353.937) | 93 | 8016.539 (53.974) | 92 | 3811.332 (38.635) | 94 | 5441.171 (48.265) | 92 | 1660.76 (15.822) | 94 | 4356.998 (31.401) | 94 | 1579.756 (14.264) | 94 | 549.138 (6.435) | 94 | 1589222.261 (13006.95) | 94 |
|  | MDD | 6941.056 (572.299) | 36 | 7899.126 (88.14) | 35 | 3745.882 (62.793) | 36 | 5371.409 (77.645) | 36 | 1628.905 (25.715) | 36 | 4353.172 (51.036) | 36 | 1586.111 (23.183) | 36 | 533.528 (10.458) | 36 | 1607308.54 (21120.33) | 36 |
| **CODE** | CTL | 7976.951 (344.051) | 74 | 8184.291 (64.931) | 74 | 3677.004 (43.3) | 74 | 5090.771 (56.463) | 74 | 1461.715 (19.857) | 74 | 4504.577 (40.154) | 74 | 1612.084 (18.493) | 74 | 483.598 (8.869) | 74 | 1548828.284 (15376.579) | 74 |
|  | MDD | 7739.908 (292.603) | 102 | 8042.78 (55.221) | 102 | 3661.025 (36.825) | 102 | 5171.045 (48.019) | 102 | 1466.824 (16.888) | 102 | 4432.727 (34.149) | 102 | 1599.804 (15.802) | 101 | 496.076 (7.58) | 101 | 1540359.872 (13078.319) | 102 |
| **DepOx** | CTL | 6664.878 (534.892) | 31 | 8675.676 (132.515) | 30 | 4048.715 (81.239) | 31 | 5478.909 (105.135) | 31 | 1746.877 (32.757) | 31 | 4124.748 (56.534) | 31 | 1385.321 (25.847) | 31 | 526.116 (15.71) | 31 | 1362278.954 (29042.624) | 31 |
|  | MDD | 6999.747 (488.697) | 37 | 8398.466 (117.587) | 38 | 4144.386 (73.267) | 38 | 5424.366 (94.818) | 38 | 1741.563 (29.542) | 38 | 4156.386 (51.682) | 37 | 1396.384 (23.311) | 38 | 527.716 (14.169) | 38 | 1310535.09 (26227.952) | 38 |
| **Imaging Genetics Dublin** | CTL | 6679.671 (332.052) | 52 | 7813.388 (109.202) | 41 | 3736.322 (53.219) | 46 | 5615.651 (80.859) | 46 | 1592.106 (22.241) | 43 | 4442.311 (44.762) | 51 | 1702.007 (24.781) | 52 | 585.418 (12.733) | 51 | 1474215.129 (27484.837) | 52 |
|  | MDD | 6445.497 (346.033) | 48 | 7699.211 (105.262) | 44 | 3767.299 (57.183) | 40 | 5759.584 (81.777) | 45 | 1595.575 (22.513) | 42 | 4385.849 (44.762) | 51 | 1734.793 (24.781) | 52 | 581.516 (12.996) | 49 | 1443020.948 (27484.837) | 52 |
| **MMDP 1.5T** | CTL | 6791.453 (410.328) | 44 | 7022.211 (92.464) | 44 | 3695.889 (62.307) | 44 | 5583.459 (96.093) | 44 | 1641.016 (31.318) | 44 | 3284.799 (57.458) | 44 | 1218.104 (33.778) | 44 | 566.467 (14.881) | 44 | 1324071.785 (16810.368) | 44 |
|  | MDD | 6956.363 (376.762) | 52 | 7170.824 (84.073) | 53 | 3642.837 (56.653) | 53 | 5768.374 (87.373) | 53 | 1595.806 (28.476) | 53 | 3309.866 (52.244) | 53 | 1274.021 (30.713) | 53 | 580.716 (13.531) | 53 | 1318317.763 (15285.375) | 53 |
| **MMDP 3T** | CTL | 6896.163 (551.065) | 63 | 7190.513 (84.787) | 64 | 3773.774 (63.633) | 64 | 5701.727 (97.508) | 63 | 1586.822 (34.388) | 64 | 4101.055 (74.291) | 64 | 1552.397 (31.698) | 63 | 687.643 (24.293) | 63 | 1334270.65 (20454.624) | 64 |
|  | MDD | 7094.725 (518.085) | 71 | 7287.834 (79.224) | 73 | 3676.387 (59.89) | 72 | 5799.168 (91.698) | 71 | 1621.507 (32.365) | 72 | 3915.255 (69.416) | 73 | 1489.517 (29.377) | 73 | 672.536 (22.841) | 71 | 1317098.334 (19114.304) | 73 |
| **MPIP** | CTL | 8818.892 (307.642) | 222 | 7073.492 (38.931) | 222 | 3523.561 (24.625) | 222 | 4799.981 (35.187) | 222 | 1424.726 (12.855) | 222 | 4157.692 (24.707) | 222 | 1300.625 (9.879) | 222 | 536.087 (5.525) | 222 | 1488615.256 (9114.622) | 222 |
|  | MDD | 9038.434 (233.636) | 368 | 7134.62 (29.566) | 368 | 3566.813 (18.701) | 368 | 4854.76 (26.723) | 368 | 1435.139 (9.762) | 368 | 4069.651 (18.793) | 367 | 1304.765 (7.502) | 368 | 534.338 (4.196) | 368 | 1519925.444 (6932.068) | 368 |
| **NESDA** | CTL | 7603.141 (424.098) | 66 | 7128.616 (77.535) | 66 | 3726.262 (46.938) | 66 | 5427.6 (59.58) | 66 | 1656.268 (20.11) | 66 | 3977.239 (43.535) | 66 | 1671.812 (20.174) | 66 | 534.181 (10.025) | 66 | 1403332.576 (21205.533) | 66 |
|  | MDD | 7737.325 (272.951) | 156 | 7140.999 (49.902) | 156 | 3645.383 (30.21) | 156 | 5314.881 (38.346) | 156 | 1663.002 (12.943) | 156 | 3869.517 (28.118) | 155 | 1623.787 (13.03) | 155 | 515.112 (6.452) | 156 | 1447890.846 (13687.025) | 156 |
| **QTIM** | CTL | 5953.172 (162.994) | 262 | 7369.803 (44.326) | 259 | 3998.126 (25.534) | 260 | 6304.493 (33.373) | 258 | 1706.745 (12.131) | 258 | 4064.651 (20.285) | 259 | 1767.142 (13.887) | 260 | 771.597 (6.508) | 260 | 1250942.494 (12205.945) | 262 |
|  | MDD | 6156.33 (430.333) | 38 | 7296.289 (116.389) | 38 | 3883.602 (68.022) | 37 | 6244.967 (87.457) | 38 | 1686.245 (31.779) | 38 | 4034.612 (54.739) | 36 | 1742.808 (36.519) | 38 | 770.1 (17.118) | 38 | 1208587.409 (32164.565) | 38 |
| **Rotterdam study** | CTL | 12852.092 (91.357) | 4356 | 6410.049 (6.77) | 4369 | 3444.598 (6.641) | 4363 | 4740.189 (7.24) | 4350 | 1540.048 (2.721) | 4372 | 4067.812 (5.103) | 4363 | 1403.836 (2.045) | 4374 | 548.964 (1.117) | 4389 | 1489885.576 (1945.914) | 4408 |
|  | MDD | 14864.288 (732.799) | 68 | 6300.27 (54.405) | 68 | 3557.156 (54.109) | 66 | 4717.23 (58.48) | 67 | 1540.473 (21.865) | 68 | 3977.368 (40.966) | 68 | 1405.81 (16.436) | 68 | 549.663 (8.994) | 68 | 1467863.014 (15585.051) | 69 |
| **SHIP** | CTL | 9728.093 (209.644) | 422 | 7102.279 (24.194) | 411 | 3578.507 (16.767) | 408 | 4855.628 (21.313) | 384 | 1552.076 (7.773) | 397 | 3904.607 (16.157) | 408 | 1480.362 (6.219) | 408 | 457.687 (2.855) | 386 | 1571014.869 (6143.991) | 441 |
|  | MDD | 9768.208 (385.635) | 129 | 7091.613 (44.867) | 123 | 3538.137 (30.413) | 128 | 4901.653 (39.029) | 117 | 1542.359 (14.463) | 119 | 3922.065 (28.503) | 135 | 1466.248 (11.229) | 129 | 459.48 (5.312) | 115 | 1590808.94 (11103.52) | 139 |
| **SHIP-trend** | CTL | 8821.668 (139.014) | 884 | 7254.004 (18.827) | 885 | 3622.718 (12.413) | 873 | 4929.271 (15.066) | 838 | 1576.587 (5.581) | 865 | 3979.707 (10.353) | 875 | 1498.57 (4.288) | 883 | 472.71 (2.086) | 798 | 1586377.419 (3941.202) | 952 |
|  | MDD | 8701.92 (239.646) | 303 | 7252.366 (32.491) | 303 | 3599.847 (21.191) | 305 | 4907.621 (26.098) | 285 | 1575.163 (9.843) | 284 | 3973.491 (17.664) | 306 | 1480.291 (7.372) | 304 | 473.698 (3.54) | 282 | 1586590.378 (6829.382) | 323 |
| **Sydney** | CTL | 7747.358 (463.926) | 106 | 7146.326 (61.633) | 106 | 4168.19 (58.437) | 106 | 6393.662 (72.337) | 106 | 1635.336 (18.616) | 105 | 4208.748 (39.453) | 106 | 1577.017 (17.808) | 106 | 532.778 (8.548) | 106 | 1499287.583 (12294.367) | 106 |
|  | MDD | 9079.575 (323.319) | 214 | 7173.986 (42.953) | 214 | 4332.366 (40.806) | 213 | 6379.102 (50.525) | 213 | 1649.354 (12.911) | 214 | 4110.809 (27.496) | 214 | 1549.776 (12.41) | 214 | 524.398 (6.006) | 210 | 1500726.805 (8568.216) | 214 |

**Supplementary Table 15:** Full results from the moderator analyses of mean age, field strength of scanner, percent of acute patients, percent of patients with anxiety, FreeSurfer version used for processing, percent of patients taking antidepressants, and percent of patients taking antipsychotics. Effect sizes for the meta-regression models were available from all 15 sites (percent of patients taking antipsychotics was not available in MMDP 1.5T and MMDP 3T). Table is split into two parts to fit on a single page for easier comparison.

A)

|  | Mean Age | | | Field Strength | | | Percent Acute | | | Percent with Anxiety | | |
| --- | --- | --- | --- | --- | --- | --- | --- | --- | --- | --- | --- | --- |
|  | Beta | Std Err | P-value | Beta | Std Err | P-value | Beta | Std Err | P-value | Beta | Std Err | P-value |
| **Lateral Ventricles** | 2.62E-03 | 3.41E-03 | 0.441 | -3.01E-02 | 7.72E-02 | 0.697 | -1.79E-03 | 1.42E-03 | 0.206 | 1.07E-04 | 2.59E-03 | 0.967 |
| **Thalamus** | 6.67E-04 | 3.51E-03 | 0.849 | 4.34E-02 | 8.01E-02 | 0.587 | -1.10E-03 | 1.75E-03 | 0.530 | 5.05E-03 | 2.85E-03 | 0.076 |
| **Caudate** | 7.46E-03 | 4.07E-03 | 0.067 | -8.47E-03 | 1.04E-01 | 0.935 | -1.00E-03 | 2.00E-03 | 0.616 | -3.92E-03 | 3.26E-03 | 0.229 |
| **Putamen** | 1.28E-03 | 3.20E-03 | 0.690 | 1.80E-03 | 7.35E-02 | 0.981 | 1.01E-03 | 1.40E-03 | 0.469 | -2.01E-03 | 2.48E-03 | 0.417 |
| **Pallidum** | 8.92E-04 | 3.09E-03 | 0.773 | -4.59E-02 | 7.07E-02 | 0.516 | -2.91E-04 | 1.40E-03 | 0.835 | -5.66E-04 | 2.48E-03 | 0.819 |
| **Hippocampus** | 1.36E-03 | 3.61E-03 | 0.707 | 1.19E-01 | 7.87E-02 | 0.130 | -4.43E-06 | 1.40E-03 | 0.997 | 1.25E-03 | 2.64E-03 | 0.638 |
| **Amygdala** | -1.52E-03 | 3.33E-03 | 0.647 | 2.81E-02 | 7.88E-02 | 0.722 | 1.59E-03 | 1.44E-03 | 0.270 | -4.45E-03 | 2.45E-03 | 0.070 |
| **Accumbens** | 2.41E-03 | 3.10E-03 | 0.436 | 7.70E-02 | 7.06E-02 | 0.276 | 3.47E-04 | 1.40E-03 | 0.804 | -2.41E-03 | 2.48E-03 | 0.330 |
| **ICV** | 6.63E-03 | 4.24E-03 | 0.118 | 1.28E-01 | 1.00E-01 | 0.201 | 2.08E-04 | 2.15E-03 | 0.923 | 5.72E-03 | 3.35E-03 | 0.088 |

B)

|  | FreeSurfer Version | | | Percent on Antidepressants | | | Percent on Antipsychotics | | |
| --- | --- | --- | --- | --- | --- | --- | --- | --- | --- |
|  | **Beta** | **Std Err** | **P-value** | **Beta** | **Std Err** | **P-value** | **Beta** | **Std Err** | **P-value** |
| **Lateral Ventricles** | -0.294 | 0.194 | 0.128 | 7.02E-04 | 1.24E-03 | 0.571 | 1.06E-02 | 5.52E-03 | 0.055 |
| **Thalamus** | 0.265 | 0.197 | 0.366 | 1.42E-03 | 1.24E-03 | 0.253 | 8.30E-03 | 6.05E-03 | 0.170 |
| **Caudate** | -0.470 | 0.257 | 0.285 | 1.48E-03 | 1.60E-03 | 0.355 | 1.61E-02 | 5.95E-03 | 6.71E-03 |
| **Putamen** | -0.187 | 0.192 | 0.135 | 9.53E-04 | 1.11E-03 | 0.392 | 1.67E-03 | 5.54E-03 | 0.763 |
| **Pallidum** | 0.039 | 0.191 | 0.979 | 5.70E-04 | 1.11E-03 | 0.608 | 4.57E-03 | 5.40E-03 | 0.397 |
| **Hippocampus** | -0.039 | 0.192 | 0.054 | -2.31E-03 | 1.12E-03 | 0.038 | -6.13E-03 | 6.25E-03 | 0.327 |
| **Amygdala** | -0.310 | 0.192 | 0.049 | 2.07E-03 | 1.10E-03 | 0.061 | 6.64E-04 | 5.78E-03 | 0.909 |
| **Accumbens** | -0.246 | 0.191 | 0.505 | -1.96E-03 | 1.11E-03 | 0.078 | -4.19E-03 | 5.39E-03 | 0.438 |
| **ICV** | 0.043 | 0.245 | 0.862 | 9.11E-04 | 1.75E-03 | 0.603 | -2.96E-04 | 8.71E-03 | 0.973 |

**Supplementary Table S16**: Meta-analytic results for each mean structure for the diagnosis * sex interactive effect in the full sample of MDD patients and Controls while controlling for age, sex, diagnosis, scan center and ICV. Adjusted Cohen's d is reported.

|  | **Cohen's d (Diagnosis * Sex)** | **Std. Err.** | **95% CI** | **P-value** | ***I*^2^** | **# Controls** | **# Patients** |
| --- | --- | --- | --- | --- | --- | --- | --- |
| **Lateral Ventricles** | -0.029 | 0.0378 | [-0.103 - 0.045] | 0.437 | 14.275 | 7058 | 1689 |
| **Thalamus** | -0.002 | 0.0463 | [-0.093 - 0.088] | 0.962 | 37.700 | 7046 | 1682 |
| **Caudate** | 0.029 | 0.0361 | [-0.042 - 0.099] | 0.428 | 8.460 | 7034 | 1681 |
| **Putamen** | -0.005 | 0.0338 | [-0.071 - 0.062] | 0.891 | 0.033 | 6957 | 1656 |
| **Pallidum** | 0.023 | 0.0484 | [-0.071 - 0.118] | 0.627 | 42.117 | 7018 | 1657 |
| **Hippocampus** | -0.008 | 0.0332 | [-0.073 - 0.057] | 0.809 | <0.001 | 7040 | 1700 |
| **Amygdala** | 0.027 | 0.0333 | [-0.038 - 0.092] | 0.413 | 0.017 | 7060 | 1696 |
| **Accumbens** | 0.029 | 0.0338 | [-0.037 - 0.095] | 0.394 | <0.001 | 6967 | 1652 |
| **ICV** | 0.002 | 0.0579 | [-0.111 - 0.116] | 0.971 | 61.128 | 7199 | 1728 |
