# Supplementary material for: Subcortical brain alterations in major depressive disorder: findings from the ENIGMA Major Depressive Disorder working group
Source: Mol Psychiatry. 2015 Jun 30;21(6):806–12. doi: 10.1038/mp.2015.69 (PMC4879183; doi:10.1038/mp.2015.69)
Supplement: Supplementary Figures [file mp201569x3.doc]

Supplemental Figures

**Supplemental Figure S1**: Overview of research institutes participating in the ENIGMA-Major Depressive Disorder Working Group, displayed on a world map.

**Supplemental Figure S2:** Forest plot of meta-analytic effect size hippocampus with *p*<0.05: MDD patients versus controls

**Supplemental Figure S3**: Forest plot of meta-analytic effect size hippocampus with *p*<0.05: Recurrent MDD patients versus controls

**Supplemental Figure S4**: Forest plot of meta-analytic effect size hippocampus with *p*<0.05: Early age of onset (≤ 21) MDD patients versus controls

**Supplemental Figure S5:** Forest plot of meta-analytic effect size amygdala with *p*<0.05: Early age of onset (≤ 21) MDD patients versus controls

**Supplemental Figure S6**: Forest plot of meta-analytic effect size lateral ventricles with *p*<0.05: Early age of onset (≤ 21) MDD patients versus controls

**Supplemental Figure S7:** Scatterplots of the percentage of patients taking antipsychotic medication versus the effect size for the caudate at each site


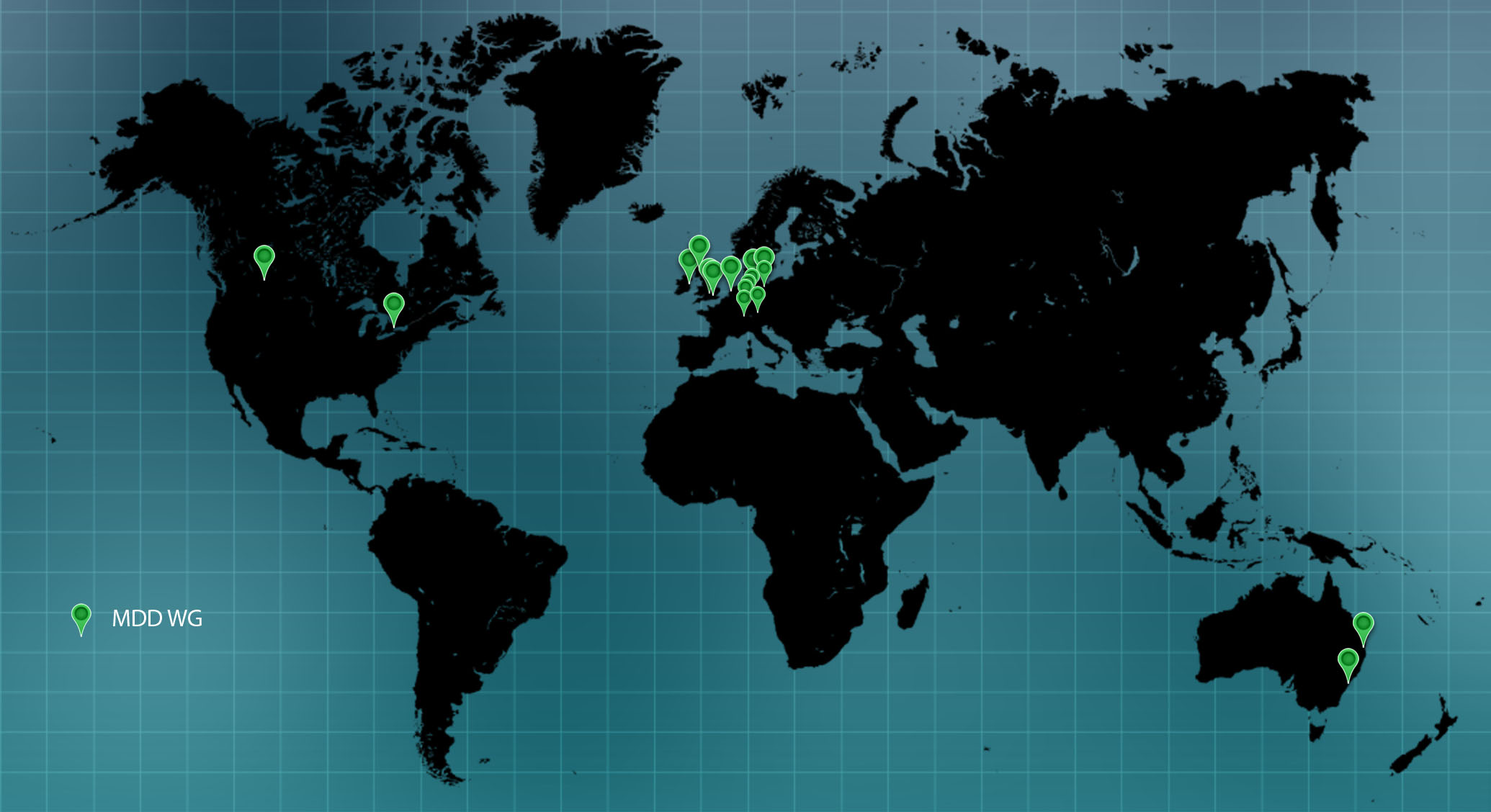
**Supplemental Figure S1**: Overview of research institutes participating in the ENIGMA-Major Depressive Disorder Working Group, displayed on a world map.


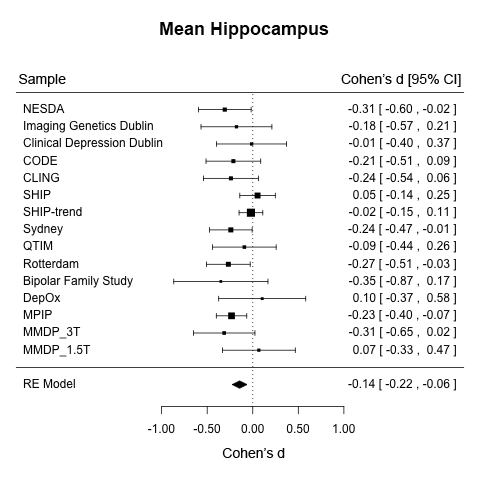


**Supplemental Figure S2:** Forest plot of meta-analytic effect size hippocampus with *p*<0.05: MDD patients versus controls


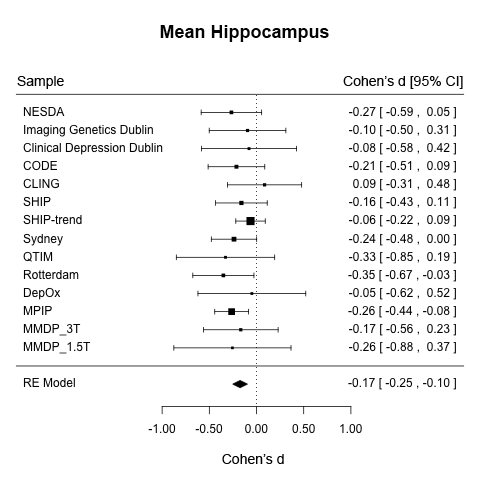


**Supplemental Figure S3**: Forest plot of meta-analytic effect size hippocampus with *p*<0.05: Recurrent MDD patients versus controls


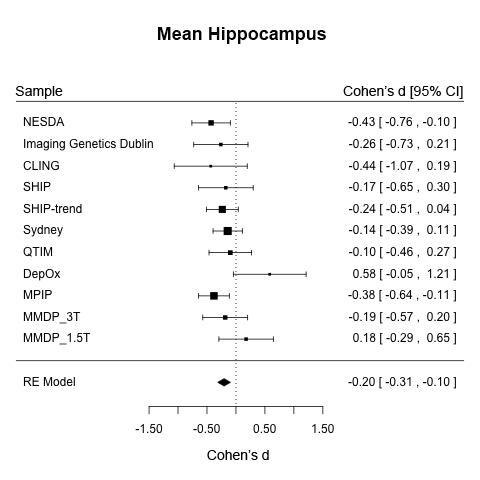


**Supplemental Figure S4**: Forest plot of meta-analytic effect size hippocampus with *p*<0.05: Early age of onset (≤ 21) MDD patients versus controls


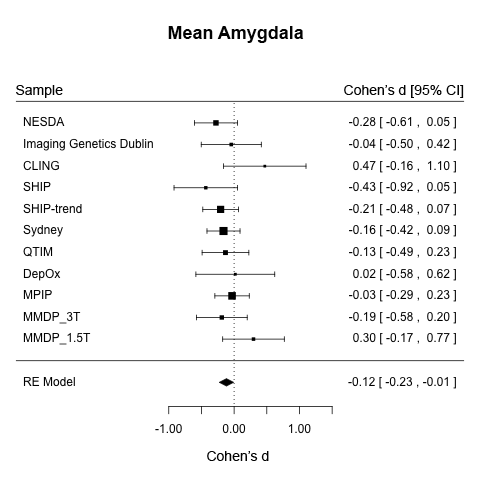


**Supplemental Figure S5:** Forest plot of meta-analytic effect size amygdala with *p*<0.05: Early age of onset (≤ 21) MDD patients versus controls


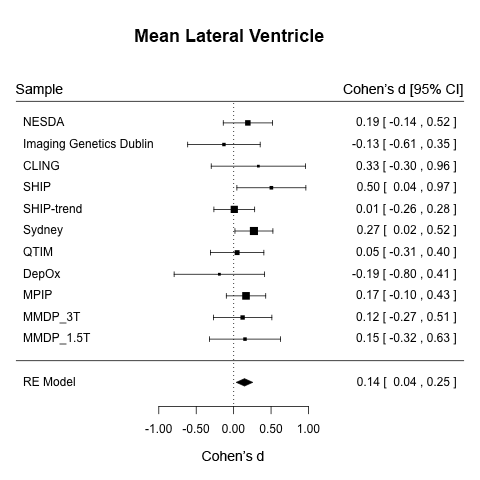


**Supplemental Figure S6**: Forest plot of meta-analytic effect size lateral ventricles with *p*<0.05: Early age of onset (≤ 21) MDD patients versus controls


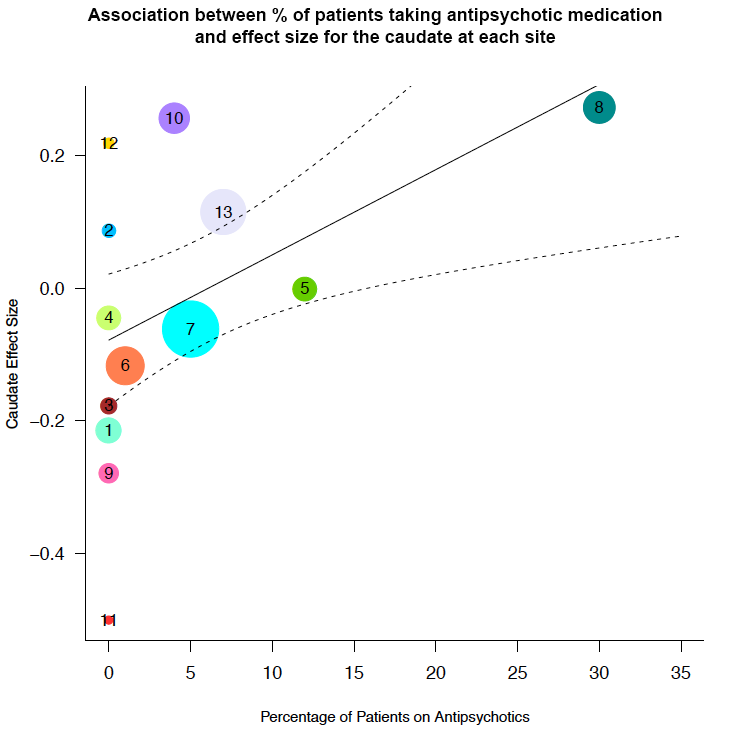


**Figure S7.** Scatterplots of the percentage of patients taking antipsychotic medication versus the effect size for the caudate at each site. Points are numbered according to the order of study sites listed in Table 1 (note: MMDP 3T and MMDP 1.5T studies were excluded from this analysis due to missing data on antipsychotic use). The size of each point corresponds to the inverse of the standard error for the effect size at each site (i.e., sites with larger samples have bigger points). The solid black line represents the effect of the percentage of patients taking antipsychotics on the effect size (the result of the moderator analysis) weighted by the inverse of the standard error in each sample. A positive association of the percentage of patients using antipsychotics on the caudate effect size was observed using a Bonferroni significance threshold for comparisons of 9 brain regions (*P** = 0.05/9 ~ 5.6x10^-3^), i.e. caudate volume of MDD patients more strongly increased relative to controls as the percentage of patients taking antipsychotic medication increased.
